# Exploring the landscape of immunotherapy approaches in sarcomas

**DOI:** 10.3389/fonc.2022.1069963

**Published:** 2023-01-09

**Authors:** Pampina Pilavaki, Myrofora Panagi, Samia Arifi, Robin L. Jones, Triantafyllos Stylianopoulos, Anastasia Constantinidou

**Affiliations:** ^1^ Medical School, University of Cyprus, Nicosia, Cyprus; ^2^ Medical Oncology, Bank of Cyprus Oncology Center, Nicosia, Cyprus; ^3^ Cancer Biophysics Laboratory, Department of Mechanical and Manufacturing Engineering University of Cyprus, Nicosia, Cyprus; ^4^ Medical Oncology Department, Hassan II University Hospital/Faculty of Medicine and Pharmacy University of Sidi Mohamed Ben Abdellah, Fez, Morocco; ^5^ Sarcoma Unit, Royal Marsden National Health Service (NHS) Foundation Trust, London, London, United Kingdom; ^6^ Sarcoma Clinical Trial Unit, Institute of Cancer Research, London, United Kingdom; ^7^ Cyprus Cancer Research Institute, Nicosia, Cyprus

**Keywords:** sarcoma, bone sarcoma, soft tissue sarcoma, immunotherapy, checkpoint inhibitors, undifferentiated pleomorphic sarcoma UPS

## Abstract

Sarcomas comprise a heterogenous group of malignancies, of more than 100 different entities, arising from mesenchymal tissue, and accounting for 1% of adult malignancies. Surgery, radiotherapy and systemic therapy constitute the therapeutic armamentarium against sarcomas, with surgical excision and conventional chemotherapy, remaining the mainstay of treatment for local and advanced disease, respectively. The prognosis for patients with metastatic disease is dismal and novel therapeutic approaches are urgently required to improve survival outcomes. Immunotherapy, is a rapidly evolving field in oncology, which has been successfully applied in multiple cancers to date. Immunomodulating antibodies, adoptive cellular therapy, cancer vaccines, and cytokines have been tested in patients with different types of sarcomas through clinical trials, pilot studies, retrospective and prospective studies. The results of these studies regarding the efficacy of different types of immunotherapies in sarcomas are conflicting, and the application of immunotherapy in daily clinical practice remains limited. Additional clinical studies are ongoing in an effort to delineate the role of immunotherapy in patients with specific sarcoma subtypes.

## 1 Introduction

Sarcomas comprise a rare and heterogenous group of malignancies, originating from mesenchymal tissue (1, 2). Among all adult malignancies, sarcomas account for approximately 1% (3). Soft tissue sarcomas (STS) and bone sarcomas (BS) are the two main categories, with an estimated incidence in Europe of 4-5 cases/100,000/year for STS and 0.8-0.9 cases/100,000/year for BS (4, 5). More than 100 different entities of sarcomas are identified; liposarcomas and leiomyosarcomas constitute the commonest STS subtypes whereas osteosarcomas represent the commonest BS subtype (4–6).

The standard of care for patients diagnosed with local/locoregional sarcomas is surgical excision of the primary tumour with clear margins (R0). Radiotherapy has a role in the treatment of non-metastatic sarcomas and it can be applied pre- or post-operatively (7–9). Additionally, neoadjuvant or adjuvant chemotherapy may be considered in selected cases of localized sarcomas, particularly if they involve patients of young age, with high grade sarcomas (4, 5). Up to 50% of patients with localized high-grade STS will eventually experience progression of disease and will develop metastases (10–13).

In the advanced setting, treatment options have not changed significantly over the last few decades, with the prognosis remaining dismal (14). The mainstay of first line treatment since the early 70s’, is anthracycline-based chemotherapy (4, 14, 15). The combination of doxorubicin with ifosfamide in the first line setting failed to demonstrate improved survival benefit over doxorubicin alone (EORTC 62012) (16). Further line treatments may include gemcitabine-docetaxel (17), high dose ifosfamide (18), dacarbazine combinations (19), trabectedin (20), eribulin (21, 22), and pazopanib (23). However, the median overall survival (OS) in metastatic disease is poor ranging from 16 to 20 months (24–26).

There is a pressing need to identify new therapeutic strategies to better manage sarcoma. Immunotherapy, is a rapidly evolving field in oncology, which has been applied with success in multiple cancers, including melanoma (27), non-small cell lung cancer (28, 29), and renal cell carcinoma (30). Novel therapeutic approaches that target the immune system including immunomodulating antibodies, adoptive cellular therapy, cancer vaccines, and cytokines have been tested in several clinical studies comprising clinical trials, pilot studies, retrospective and prospective studies in patients with different types of sarcomas. Although the use of immunotherapy in daily clinical practice in sarcomas remains minimal, certain sarcoma subtypes may benefit from this approach. The challenge ahead, for the sarcoma research community and the industry, is to identify these subtypes and predictive biomarkers. This review provides a thorough documentation of the clinical landscape of past and current efforts to explore the role of immunotherapy in sarcomas.

## 2 Overview of cancer immunotherapy

The critical dual role of the immune system in the elimination and development of cancer has been well established and is known as “cancer immunoediting”. The concept of cancer immunoediting consists of the elimination, equilibrium and escape phases. Specifically, through the elimination process the potentially malignant cells are identified and decimated by the innate and adaptive immune system. The elimination phase is followed by the equilibrium phase, considered to be the longest of all three phases, lasting possibly for years. Tumour cells that have not been destroyed during the elimination process enter the equilibrium phase where their expansion is prevented and tumour cells immunogenicity takes place. Subsequently, tumour cells evade the immune system leading to the escape phase where uncontrolled proliferation occurs (31, 32).

Cancer immunotherapy aims to manipulate and stimulate the immune system against malignant cells (33, 34). In order to achieve this, multiple strategies and forms of immunotherapy have been proposed. Immunotherapy might affect any of the three phases of immunoediting, leading eventually to response to treatment and elimination of the tumour cells or disease may remain in a dormant status as per the equilibrium phase or even progression may be observed as part of the escape process (32).

Immunotherapy can be further categorized into passive and active therapy. Passive immunotherapy describes the delivery of effector modules or cells to the patient to enrich the existing anti-neoplastic activity (35, 36). It includes the use of immunomodulating antibodies (immune co-stimulatory antibodies or immune checkpoint inhibitors) or the use of adoptive immunotherapy (36). Additionally, adoptive immunotherapy includes the production of genetically modified blood-derived T cells (genetically engineered T-cell receptors (TCR) and Chimeric Antigen Receptor CAR-T cells), or the isolation of tumour infiltrating lymphocytes (TILs) from the tumour microenvironment followed by ex vivo expansion and subsequent administration to the patient (35, 36). On the other hand, active immunotherapy aims to activate the patient’s immune system in order to assail and defeat malignant cells (36). Cancer vaccines and cytokines administration compose the group of active immunotherapies (35, 36).

## 3 Materials and methods

We conducted a narrative review searching in PubMed for articles published from inception to November 2022 using the keywords “immunotherapy” AND (“bone sarcoma” OR “soft tissue sarcoma”). Original clinical studies including clinical trials, pilot studies, prospective studies, and retrospective studies that have enrolled at least one patient diagnosed with sarcoma and treated with immunotherapy, and published in English language, are included in this review article. Furthermore, we searched and included clinical studies that were presented in abstract form at the European Society of Medical Oncology (ESMO), the American Society of Clinical Oncology (ASCO), or the Connective Tissue Society Oncology (CTOS) meetings during this time. In total of 92 studies have been identified and are presented in **Tables S1–S4** (**Supplementary Material**). The different types of immunotherapies that have been investigated in selected histological types of STS and BS are presented in **Tables 1**, **2**.

**Table 1:** Different types of immunotherapies that have been investigated in selected histological types of STS.

Type of sarcoma	Immunomodulating antibodies	ACT	Vaccines	Cytokines
Leiomyosarcoma	-Nivolumab (37–42) -Nivolumab & Ipilimumab (38–40, 43, 44) -Nivolumab & Bempegaldesleukin (45) -Ipilimumab (46) -Pembrolizumab (40, 47–56) -Sintilimab (57) -Durvalumab & Tremelimumab (58) -Avelumab & Trabectedin (59)		-DC-based vaccines (60, 61) -Peptide-based vaccines (62) -T-VEC + pembrolizumab (63)	
Liposarcoma	-Nivolumab (38, 39, 41) -Nivolumab & Ipilimumab (38, 39, 43, 44) -Nivolumab & Bempegaldesleukin (45) -Pembrolizumab (37, 47–51, 53–56, 64) -Spartalizumab (65) -Sintilimab (57) -Durvalumab & Tremelimumab (58) -Avelumab & Trabectedin (59)	-NY-ESO-1–specific ETC (66) -ADP-A2M4 (67) -Transgenic ACT with DC vaccination ± ipilimumab (68) -Letetresgene autoleucel (autologous T-cell therapy targeting NY-ESO-1 tumours) (69)	-CMB305 (Lentiviral-Based) prime -boost vaccine (70) -DC-based vaccines (60) -Peptide-based vaccines (62, 71)	-IFNγ (72)
Undifferentiated pleomorphic sarcoma/Malignant fibrous histiocytoma	-Nivolumab (38, 41, 73) -Nivolumab & Ipilimumab (38, 40) -Nivolumab & Bempegaldesleukin (45) -Pembrolizumab (40, 47–56, 64) -Pacmilimab -Sintilimab (57) -Durvalumab & Tremelimumab (58)		-DC-based vaccines -Peptide-based vaccines (62) -T-VEC + pembrolizumab (63)	
Synovial sarcoma	-Nivolumab (38, 41, 73) -Nivolumab & Ipilimumab (38, 43, 44) -Nivolumab & Bempegaldesleukin (45) -Ipilimumab (74) -Pembrolizumab (51) -Sintilimab (57) -Durvalumab & Tremelimumab (58)	-NY-ESO-1–specific ETC (66) - NY-ESO-1 SPEAR T-cells (75) -Autologous TCR-transduced T cells (against NY-ESO-1) (76) -Autologous CD4+ T-cells transduced with MAGE-A3 TCR (77) -PHA activated autologous PBL (78) -ADP-A2M4 (67, 79) -Transgenic ACT with DC vaccination ± ipilimumab (68)	-CMB305 (Lentiviral-Based) prime -boost vaccine (70) -DC-based vaccines (60, 80–82) -Peptide-based vaccines (62) -T-VEC + pembrolizumab (63)	-IFNγ (72)
Spindle cell sarcoma	-Nivolumab (38) -Nivolumab & Ipilimumab (38) -Pembrolizumab (37, 48)			
Alveolar soft part sarcoma	-Nivolumab (38, 41, 73, 83) -Nivolumab & Ipilimumab (38) -Nivolumab & Bempegaldesleukin (45) -Pembrolizumab (37, 48, 53, 54, 84) -Geptanolimab (85) -Toripalimab -Atezolizumab (86) -Durvalumab & Tremelimumab (58)		-DC-based vaccines (60) -Peptide-based vaccines (62) -T-VEC + pembrolizumab (63)	
Epithelioid sarcoma	-Nivolumab (38, 41, 53, 73) -Pembrolizumab (37, 48, 50) -Ipilimumab (38) -Sintilimab (57)	-PHA activated autologous PBL (78)	-Peptide-based vaccines (62) -T-VEC + pembrolizumab (63)	
Malignant solitary fibrous tumour	-Nivolumab (38, 73) -Pembrolizumab (48) -Nivolumab & Ipilimumab (43) -Pembrolizumab (49)			
Malignant peripheral nerve sheath tumour	-Nivolumab (38, 41) -Nivolumab & Ipilimumab (38, 43, 44) -Pembrolizumab (53) -Ipilimumab (46)	-Transgenic ACT with DC vaccination ± ipilimumab (68)	-DC-based vaccines (60) -T-VEC + pembrolizumab (63)	
Angiosarcoma	-Nivolumab (73) -Nivolumab & Ipilimumab (38, 43, 87, 88) -Nivolumab & Bempegaldesleukin (45) -Pembrolizumab (37, 48, 49, 51, 53, 55, 88) -Carotuximab (89) -Sintilimab (57) -Durvalumab & Tremelimumab (58)		-DC-based vaccines (60) -T-VEC + pembrolizumab (63)	-rIL-2 (90–93)
Myxofibrosarcoma	-Nivolumab & Ipilimumab (38, 43, 44) -Pembrolizumab (37, 48–50, 55)		-T-VEC + pembrolizumab (63)	
Rhabdomyosarcoma	-Pembrolizumab (37, 48, 50, 51, 53, 94) -Nivolumab (41) -Nivolumab & Ipilimumab (43) -Sintilimab (57)	-NK-92 cells (95)	-DC-based vaccines (80, 81, 96)	
Clear cell sarcoma	-Nivolumab (73, 83) -Pembrolizumab (53, 56, 94) -Ipilimumab (46) -Camrelizumab (97)		-DC-based vaccines (60, 98) -Peptide-based vaccines (62)	
Fibrosarcoma	- Nivolumab & Ipilimumab (99) -Sintilimab (57)	-PHA activated autologous PBL (78)	-DC-based vaccines (98)	
Dermatofibrosarcoma protuberans	-Nivolumab (41) -Nivolumab & Ipilimumab (43)			
Desmoplastic small round cell tumour	-Nivolumab (41) -Pembrolizumab (50, 53, 84)	-HER2-specific CAR T cell (100)	-DC-based vaccines (80, 98)	
Epithelioid Hemangioendothelioma	-Nivolumab (73) -Pembrolizumab (48, 55)			
Endometrial stromal sarcoma	-Pembrolizumab (48, 49)	-Autologous ex vivo-generated anti-tumour-specific CTL (101)		
Hemangiopericytoma	-Pembrolizumab (48)			
Histiocytic sarcoma	-Nivolumab (37)			
Sarcoma, not otherwise specified	-Nivolumab (38) -Nivolumab & Ipilimumab (38, 43, 44) -Ipilimumab (46)			
Intimal sarcoma	-Nivolumab (41)			
Ossifying fibromyxoid tumour	-Pembrolizumab (37)			
Fibromyxoid sarcoma	-Pembrolizumab (50, 51, 56)			
Extraskeletal myxoid chondrosarcoma	-Nivolumab (38, 73, 102) -Nivolumab & Ipilimumab (38) -Pembrolizumab (37, 48, 49)		-T-VEC + pembrolizumab (63)	
Extraskeletal osteosarcoma	-Pembrolizumab (37, 51)			

ICI, immune checkpoint inhibitors; ACT, adoptive cell therapy; IT, immunotherapy; ETC, endogenous T cells; CTL, cytotoxic T lymphocytes; TCR, T-cell receptor; CAR, Chimeric Antigen Receptor; NK, natural killer; PHA, phytohemagglutinin; PBL, peripheral blood lymphocytes; rIL-2, recombinant interleukin-2; T-VEC, Talimogene laherparepvec.

**Table 2:** Different types of immunotherapies that have been investigated in selected histological types of BS.

Type of sarcoma	ICI	ACT	Vaccines	Cytokines
Chondrosarcoma	-Nivolumab (37, 38, 41, 73) -Nivolumab & Ipilimumab (38) -Nivolumab & Bempegaldesleukin (45) -Pembrolizumab (37, 47, 48, 53) -Atezolizumab (103)		-DC-based vaccines (60) -Peptide-based vaccines (62) -T-VEC + pembrolizumab (63)	
Osteosarcoma	-Nivolumab (38, 41, 73) -Nivolumab & Ipilimumab (38, 43) -Nivolumab & Bempegaldesleukin (45) -Pembrolizumab (37, 47, 94, 104) -Durvalumab & Tremelimumab (58)	-TILs therapy (105) -Autologous CD4+ T-cells-transduced with MAGE-A3 TCR (77) -HER2-specific CAR T cell (100) -NK-92 cells (95) -PHA activated autologous PBL (78) -Transgenic ACT with DC vaccination ± ipilimumab (68) -TILs + Nivolumab (106, 107)	-DC-based vaccines (60, 108) -Peptide-based vaccines (62) -Irradiated autologous tumour cells vaccine (109)	-IL-2 (110)
Ewing Sarcoma	-Nivolumab (38) -Nivolumab & Ipilimumab (38, 47) -Pembrolizumab (49, 94)	-HLA-A*02:01/peptide-specific allorepertoire-derived CD8^+^ T cells (111) -HER2-specific CAR T cell (100)	-DC-based vaccines (60, 80–82, 96, 98, 108) -Vigil vaccine (112) -Intratumoral Pexa-Vec (JX-594) (113)	
Chordoma	-Nivolumab (37) -Pembrolizumab (37, 84) -Ipilimumab (46) -Durvalumab & Tremelimumab (58)		-Yeast-Brachyury Vaccine (GI-6301) (80)	

ICI, immune checkpoint inhibitors; ACT, adoptive cell therapy; IT, immunotherapy; TILs, tumour-infiltrating lymphocytes; TCR, T-cell receptor; CAR, Chimeric Antigen Receptor; NK, natural killer; PHA, phytohemagglutinin; PBL, peripheral blood lymphocytes; Pexa-Vec, pexastimogene devacirepvec; IL-2, interleukin-2; T-VEC, Talimogene laherparepvec.

## 4 Clinical experience with passive immunotherapy in sarcomas

### 4.1 Immunomodulating antibodies and combination therapies

**Figure 1:**
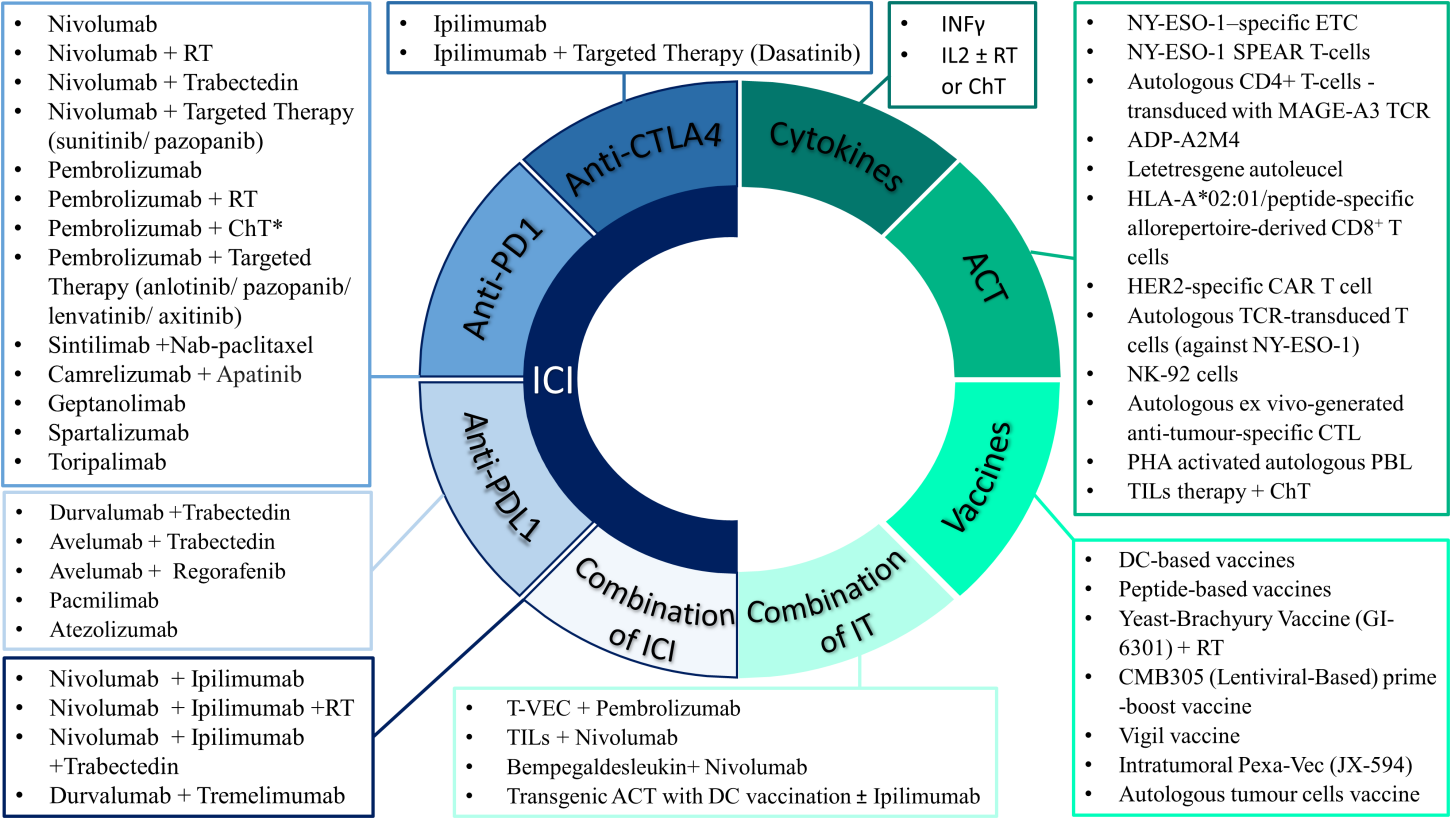
Selected approved and experimental immunotherapy drugs that have been used as single agent or in combination with other treatment modalities in patients with sarcomas. *ChT includes the following regimens: Doxorubicin, AIM (anthracycline, ifosfamide, mesna), AD (anthracycline, dacarbazine), High-dose ifosfamide, Gemcitabine ± Docetaxel or Dacarbazine or Nab-paclitaxel or Vinorelbine, Irinotecan, Liposomal doxorubicin, Metronomic cyclophosphamide. IT, immunotherapy approaches; ICI, immune checkpoint inhibitors; CTLA4, cytotoxic T lymphocyte-associated molecule 4; PD1 programmed death 1; PDL1, programmed death-ligand 1; ACT, adoptive cell therapy; ETC, endogenous T cells; CTL, cytotoxic T lymphocytes; TCR, T-cell receptor; CAR, Chimeric Antigen Receptor; TILs, tumour-infiltrating lymphocytes; NK, natural killer; PHA, phytohemagglutinin; PBL, peripheral blood lymphocytes; IL-2, interleukin-2; ChT, chemotherapy; RT, radiotherapy; T-VEC, Talimogene laherparepvec; Pexa-Vec, pexastimogene devacirepvec.

Identification of immune checkpoint molecules including CTLA-4 (cytotoxic T lymphocyte-associated molecule 4) and PD-1 (programmed cell death 1) T-cell surface molecules, led to the development of immune checkpoint inhibitors and the subsequent suppression of the associated inhibitory pathways (36). Ipilimumab, an anti-CTLA-4 antibody, was the first immune checkpoint inhibitor to be approved by FDA in 2011 for the treatment of metastatic melanoma, followed by several other immune checkpoint inhibitors with multiple clinical indications (114). Several clinical studies have investigated the use of checkpoint inhibition in STS or BS (summarized in **Table S1**, **Supplementary Material**). Contrary to melanoma however, the mutational burden of sarcomas is low, the numbers of TILs in the tumour microenvironment are low and the PD-L1+ expression is significantly lower, which may explain the variable responses documented with different immune checkpoint inhibitors. Anti- PD-1 agents (pembrolizumab, nivolumab, geptanolimab, spartalizumab, toripalimab, camrelizumab, sintilimab), anti- PD ligand 1 (PD-L1) agents (durvalumab, pacmilimab, avelumab, atezolizumab), and anti-CTLA4 (ipilimumab, tremelimumab) agents have been used as single therapy or combined with chemotherapy or targeted therapy or other types of immunotherapies (**Figure 1**).

#### 4.1.1 Immune checkpoint inhibitor monotherapy

Single agent treatment responses have in general been disappointing with exceptional effectiveness reported in certain subtypes such as alveolar soft part sarcoma (85, 86). One of the early trials - published in 2017 - that actually demonstrated single agent activity was SARC028 a non-randomized, open-label, single arm, two cohort, phase 2 clinical trial of Pembrolizumab (200mg IV 3 weekly), in which 86 patients with STS or BS were enrolled (47). Eighty patients with at least one and up to 3 previous lines of therapy, were evaluable for response. The STS cohort included patients with undifferentiated pleomorphic sarcoma (UPS), liposarcoma, synovial sarcoma, and leiomyosarcoma (10 patients of each histological type), whilst 40 patients were included in the BS cohort which consisted of osteosarcoma, Ewing sarcoma, and dedifferentiated chondrosarcoma. The objective response rate (ORR) was 18% for the STS cohort and 5% for the BS cohort. However, significant variability was observed across different histological types in the STS cohort. One complete response (CR) and 3 partial responses (PR) were documented in the UPS cohort (ORR 40%). The study reported 2 PRs in patients with liposarcoma, 1 PR in synovial sarcoma and none in patients with leiomyosarcoma. Regarding the BS cohort, 1 out of 22 patients with osteosarcoma and 1 out of 5 patients with chondrosarcoma experienced PR, and none of the 13 patients with Ewing sarcoma. The median duration of response was 33 weeks (IQR, 23–49) in the STS cohort, with ongoing responses at the analysis time, and 43 weeks (IQR, 25–61) for the BS cohort. Additionally, the median progression free survival (PFS) was 18 weeks (95% CI 8-21) for the STS patients and 8 weeks (95% CI 7-9) for the BS patients; the 12-weeks PFS rate was 55% (95% CI 40-70) for the STS population. Of note, for UPS and liposarcoma the median PFS was estimated at 30 weeks (95% CI 8-68) and 25 weeks (95 CI 8-42), respectively. The 12-weeks PFS rate was 70% (95% CI 42-98) for UPS and 60% (95% CI 30-90) for liposarcoma. The median OS was 49 weeks (95% CI 34-73) for the STS cohort and 52 weeks (95% CI 40-72) for BS cohort. Interestingly, the pre- treatment PD-L1 expression levels were examined in 70 tissues and only 3 had tested positive, all of them being from patients with UPS; specifically, one achieving CR, the second one PR and the third one not being evaluable. Regarding toxicity, fatigue, anaemia, and lymphopenia were the most common grade 3-4 adverse events, while 9 patients developed serious adverse events including adrenal insufficiency, pneumonitis, and interstitial nephritis. Overall, the conclusion was that pembrolizumab demonstrated promising efficacy in patients with UPS and liposarcoma therefore warranting further investigation (47). Thus, expansion of these two cohorts (UPS and liposarcoma) was performed and their results were presented in ASCO annual meeting in 2019 (64). Additional 30 patients with UPS and 30 with dedifferentiated/pleomorphic liposarcoma were enrolled, leading to 40 patients in total in each cohort. The ORR was 23% (9/40) and 10% (4/39 evaluable patients) in UPS and liposarcoma cohort, respectively. The median PFS was 3 months (95% CI 2-5) and the median OS was 12 months (95% CI 7-34) for UPS patients, whilst for liposarcoma the median PFS was 2 months (95% CI 2-4) and the median OS was 13 months (95% CI 8-NR) (64). These data indicate that PD1 inhibition has meaningful activity in a small subset of patients with STS, and predictive biomarkers are needed to better select patients that are likely to respond. Studies examining single agent activity assessed not only pembrolizumab, nivolumab and ipilimumab but also the role of newer molecules including pacmilimab and geptanolimab (37, 83–85, 94, 115, 116).

#### 4.1.2 Dual checkpoint inhibitors

. Due to the limited activity of single agent checkpoint inhibitors, combination immunotherapies were explored. In particular, nivolumab plus ipilimumab demonstrated preliminary activity in STS. The Alliance A091401 trial, a non-comparative, multicentre, open-label, unblinded, randomized phase 2 clinical trial that included patients from 15 centres in the USA, investigated the use of nivolumab versus nivolumab plus ipilimumab in patients with sarcoma (BS or STS) and published its first results in 2018 (38). The one treatment arm consisted of nivolumab 3mg/kg every 2 weeks, and the other consisted of nivolumab 3mg/kg plus ipilimumab 1mg/kg every 3 weeks for 4 doses and then nivolumab 3mg/kg every 2 weeks. Among the 85 enrolled patients with metastatic or unresectable sarcoma, 76 were evaluable for efficacy; 38 received nivolumab and 38 patients received combined treatment with nivolumab plus ipilimumab. The primary endpoint, ORR, was 5% for nivolumab monotherapy, and 16% with combined therapy in patients with UPS, leiomyosarcoma (uterine and non-uterine), myxofibrosarcoma and angiosarcoma among the patients treated with nivolumab plus ipilimumab. As per secondary endpoints, in the monotherapy arm the 6-month clinical benefit rate was 10% and the 12-months clinical benefit rate was 2%, compared to 12% 6-month clinical benefit rate and 12% 12-months clinical benefit rate in the combination therapy arm. Additionally, the median PFS was 1.7 months and 4.1 months in the monotherapy arm and the combination therapy arm, respectively. Also, the median OS was estimated at 10.7 months for the monotherapy and 14.3 months for the combination therapy. Overall, the results of this trial suggested that nivolumab as a single agent has limited efficacy in unselected sarcoma patients, while the combination of nivolumab and ipilimumab showed more encouraging results in specific subtypes of sarcomas with tolerable toxicity, therefore further studies are required to define its exact role.

Of note, results of 3 expansion cohorts of the Alliance A091401 trial (NCT02500797) were presented in the ASCO annual meeting in 2020 (117). The cohorts consisted of gastrointestinal stromal tumour, UPS, or dedifferentiated liposarcoma, and the primary endpoint was the assessment of 6-month confirmed RR. The primary endpoint was met for the UPS and liposarcoma cohorts but only for the combination therapy with nivolumab and ipilimumab (RR 14% for UPS, and 14% for dedifferentiated liposarcoma), not for the therapy with single agent nivolumab (RR 8% for undifferentiated pleomorphic sarcoma, and 7% for dedifferentiated liposarcoma). The trial is still in progress, in active not recruiting status.

The comparison of nivolumab versus the combination ipilimumab/nivolumab was examined in the neoadjuvant setting in patients with surgically resectable retroperitoneal dedifferentiated liposarcoma or extremity/truncal UPS receiving concomitant neoadjuvant radiotherapy. The preliminary results of a phase 2 clinical trial in currently active, not recruiting status (NCT03307616) were reported in 2020 (39). In particular, 24 patients were evaluable for response and the median pathological response (primary end point) was 95% and 22.5% in patients with UPS and dedifferentiated liposarcoma, respectively; providing encouraging preliminary results for combination radiation therapy and immune checkpoint inhibitors. Additionally, the SU2C-SARC032 (NCT03092323), a randomized, phase 2, currently in recruiting status trial investigates the combination of neoadjuvant pembrolizumab and radiotherapy followed by surgery and adjuvant pembrolizumab versus neoadjuvant radiotherapy followed by surgery in high-risk localized soft tissue sarcoma of the extremity (118).

The combination was also examined in angiosarcoma, a disease with very limited systemic therapeutic options available. In a phase 2, open-label, multicentre clinical trial including patients with metastatic or unresectable angiosarcoma, the combination of ipilimumab (1mg/kg every 6 weeks) with nivolumab (240mg every 2 weeks) was explored in 16 patients. The results reported by Wagner MJ et al. (87) showed ORR at 25% (4/16), and a 6-month PFS (secondary endpoint) at 38%. Interestingly, 60% of the patients with primary cutaneous scalp or face angiosarcoma experienced objective response to treatment. Results that indicated additional investigation of the combination of ipilimumab and nivolumab in patients with angiosarcoma, may be worth performing. Clinical activity of immune checkpoint inhibitors in patients with angiosarcomas has also been reported in a retrospective case series study, including 7 patients treated off label or in the context of clinical trial with pembrolizumab (single agent or combined with axitinib) or AGEN1884 (a CTLA-4 inhibitor) or ipilimumab and nivolumab (88).

Retrospective studies have also reported on the combination of nivolumab with ipilimumab concluding that the combination is associated with manageable toxicity and could be effective in certain types of sarcomas in the advanced disease (40, 43, 99).

#### 4.1.3 Combinations of immune checkpoint inhibitors with chemotherapy

Combinations of immunotherapy with chemotherapy have provided mixed results. Cytotoxic chemotherapy due to its potential immunogenic effects (including suppressive immune cells depletion or inhibition, release of damage-associated molecular patterns and increased tumour antigen presentation), may synergize with immunotherapy leading to increased effectiveness (48, 119, 120). Currently, the combination of immunotherapy with chemotherapy is being applied in clinical practice in other malignancies including non-small-cell lung cancer (121) and has been under investigation in sarcomas.

One of the most important prospective trials in this category, the PEMBROSARC was a multicohort phase 2 study of pembrolizumab (200 mg IV) combined with low-dose cyclophosphamide (50 mg twice daily; 1 week on followed by 1 week off). One of its strata included 17 adults diagnosed with metastatic and/or unresectable osteosarcomas 15 of which were evaluated for efficacy (104). The non-progression and objective responses at 6 months as per RECIST 1.1 were the primary endpoints of the trial. The results revealed that only one patient experienced PR (6.7%), and that participant did not express PD-L1. Five patients (33.3%) had stable disease (SD), and 8 patients (53.3%) experienced progression. Among the 14 participants who were tested for PD-L1 expression, only 2 were positive. The 6-month non-progression rate was 13.3%, the median PFS was 1.4 months (95% CI = 1.0 months - 1.4 months), and the median OS was 5.6 months (95% CI = 2.1 months - 12.1 months). Results suggested limited activity of PD-1 inhibitors in patients with advanced osteosarcoma.

The results of another strata of the PEMBROSARC trial including patients with STS, were reported earlier by Toulmonde M et al. (49). Fifty-seven patients were enrolled in the study; 15 with leiomyosarcoma, 16 with UPS, 10 with gastrointestinal stromal tumour and 16 with other types of sarcomas. The 6-month non-progression rate was 0% for both leiomyosarcoma and UPS, 14.3% for other sarcomas and 11.1% for gastrointestinal stromal tumour. PR was reported in only one patient who was also the only one with strong PD-L1–positive staining. In the majority of patients, strong infiltration of macrophage expressing the IDO1 (inhibitory enzyme indoleamine 2,3-dioxygenase) was reported. A limited efficacy of PD-1 inhibitors in certain sarcoma types was observed, which was attributed to macrophage infiltration, IDO1 activation and subsequent immunosuppressive tumour microenvironment.

Subsequently, in June 2022 the results of a biomarker-driven cohort of the PEMBROSARC trial were reported (50). Thirty patients selected based on the presence of intratumoral tertiary lymphoid structures (TLSs) and treated with low-dose cyclophosphamide and pembrolizumab were included in the efficacy analysis. The 6-month non-progression rate was 40% (95% CI, 22.7-59.4) and the ORR was 30% (95% CI, 14.7–49.4). Specifically, 9 patients experienced PR, 10 patients had SD, and 10 had PD. Additionally, the median duration of response was 11 months (95% CI, 1.1 months to not attained), the median PFS was 4.1 months (95% CI, 1.4–12.5 months), and the median OS was 18.3 months (95% CI, 8.5 months to not attained). With regards to toxicity, grade 1 or 2 fatigue, nausea, dysthyroidism, diarrhoea and anaemia were the most frequent toxicities. Interestingly, abundance of intratumoral plasma cells was correlated with improved outcome. Overall, this biomarker-driven cohort demonstrated better results in terms of efficacy compared to other cohorts of PEMBROSARC trial, and the presence of TLSs in advanced STS is suggested as a potential predictive biomarker to support patient selection for treatment with immune checkpoint inhibitors. Importantly, the impact of immunotherapy compared to standard chemotherapy in patients with TLS-positive sarcomas is now further evaluated through randomized phase 2 trials, in recruiting status, in both the neoadjuvant setting (NCT04968106) and the metastatic setting (NCT04874311).

The combination of pembrolizumab with doxorubicin have also been tested in clinical trials (48, 51). A phase 1/2 non-randomized trial reported by Pollack SM et al. evaluating the safety and efficacy of pembrolizumab combined with doxorubicin in advanced anthracycline-naïve sarcomas (48). Safety of doxorubicin was tested in two dose levels (45 and 75 mg/m^2^) whilst the recommended dose for phase 2 was determined at 75 mg/m^2^. Thirty-seven patients were treated in the combined phase 1/2 and the ORR (primary endpoint) was 13% for phase 2 and 19% overall. Durable PRs were observed in 2 of 3 patients with UPS and 2 of 4 patients with dedifferentiated liposarcoma. The median PFS and the median OS were 8.1 (95% CI 7.6-10.8) months and 27.6 (95% CI 18.7-NR) months, respectively. Immunohistochemistry was applied in 29 patients, and 66% had PD-L1 expression scores of 0 and there was no association of PD-L1 expression with PFS and OS. Additionally, TILs were presented in 21% of assessable tumours and correlated with inferior PFS. Another phase 2, single arm study assessed safety (primary endpoint) and efficacy of pembrolizumab with doxorubicin (60 mg/m^2^ cycle 1 with escalation to 75 mg/m^2^ on cycle 2) in advanced anthracycline-naive STS (51). Thirty patients were enrolled, and the ORR was 36.7%. One patient with liposarcoma (3.3%) achieved CR, 10 patients (33.3%) had PR, and 13 patients (43.3%) had SD. The median PFS was 5.7 months (95% CI 4.1-8.9) and the median OS was 17 months (95% CI 9.9-NR). Interestingly, PD-L1 expression was associated with improved ORR but there was no correlation with PFS and OS. Both studies demonstrated manageable toxicity profile. The results of the phase 1/2 and phase 2 of SAINT clinical trial (NCT03138161) which is currently on recruiting status, were presented in the ASCO’s annual meeting in 2019 and subsequently in 2020 and in 2022 (122–124). In this study, untreated patients with locally advanced or unresectable or metastatic STS were treated with combination treatment with ipilimumab, nivolumab, and trabectedin. The recommended doses for phase 2 were ipilimumab 1 mg/kg IV q 12 weeks, nivolumab 3 mg/kg IV q 2 weeks, and trabectedin 1.2 mg/m2 IV q 3 weeks. The ORR was 21.6%, and the disease control rate was 87.5% among the 88 evaluable for efficacy patients in phase 2. The median OS and the median PFS were 14 months and 7 months, respectively. These results suggested that the combination may have clinical benefit in sarcoma. Another phase 1/2 trial published in June 2022 investigated the combination of an immune checkpoint inhibitor with trabectedin (59). This single arm, open label trial tested the safety and efficacy of avelumab (anti-PD-L1 antibody) combined with trabectedin in patients with advanced leiomyosarcoma (18 patients with non-uterine and 6 with uterine disease) and liposarcoma (11 patients). The recommended phase 2 dose was determined at 1.0 mg/m^2^ for trabectedin and 800mg for avelumab. Twenty-three patients were evaluable at recommended phase 2 dose. PR was reported in 3 patients (13%), and SD in 10 patients (43%). The six-month PFS and the median PFS were 52% and 8.3 months, respectively.

Several other studies currently on active, not recruiting status, have reported their preliminary results on the combination of chemotherapy with immunotherapy, over the last few years. The TRAMUNE trial (NCT03085225) a phase 1b study reported its results regarding safety and preliminary efficacy initially at the 2020 ESMO Congress and was subsequently published in May 2022 (125, 126). Patients with unresectable or metastatic STS and relapsed ovarian carcinoma were treated with trabectedin and durvalumab. Nine patients were enrolled in the 3 + 3 dose escalation phase (1 mg/m^2^, 1.2 mg/m^2^, 1.5 mg/m^2^ dose levels of trabectedin given on day 1, combined with durvalumab 1120 mg/m^2^ on day 2 q 2 weeks), 16 patients were included in the STS cohort and 15 in the ovarian carcinoma cohort. Recommended phase 2 dose and ORR were the primary endpoints. The maximum tolerated dose was defined as 1.2 mg/m2 and 1120 mg/m2 for trabectedin and durvalumab, respectively. Regarding the STS cohort, 16 patients were evaluable for safety and 14 for efficacy. Efficacy wise, 1 PR was reported corresponding to 7.1% (CI95% 0.2 - 33.9) ORR, and the 6-month progression free rate was estimated at 28.6% (CI95% 8.4 - 58.1). Additionally, tumour shrinkage was observed in 43% of the patients. As per toxicity, 50% (8 patients) experienced drug-related grade 3/4 adverse events including neutrophil count decrease in 35.7%, and 2 patients had grade 5 adverse events including multi-organ failure and febrile aplasia.

Furthermore, the phase 2 clinical trial (NCT03899805) which investigated the combination of eribulin and pembrolizumab in patients diagnosed with metastatic STS presented the results of the leiomyosarcoma cohort in 2020 (127). The estimated PFS at 12 weeks (primary endpoint) was 42.1% which did not meet the predefined endpoint. In addition, the phase 1 clinical trial (NCT03123276) using gemcitabine and pembrolizumab in patients with advanced leiomyosarcoma and UPS was presented in the 2021 ESMO congress (52). Two patients with UPS and 11 with leiomyosarcoma were included, and a 3 + 3 design was applied using 800 mg/m2, 1000 mg/m2, 1200 mg/m2 of gemcitabine. The maximum tolerated dose was not reached and the recommended dose of gemcitabine was 1200 mg/m2. Sixteen serious adverse events were reported with fever to be the most frequent. The median PFS was estimated at 5.1 months, and 92% of the patients were free of progression at 9 weeks.

Retrospective analyses have also suggested activity of immunotherapy combinations with chemotherapy in certain sarcoma subtypes. Liu J et al. (53), reported on the combination of pembrolizumab with either chemotherapy or targeted agents (anlotinib 12 mg/day or pazopanib 400–600 mg/day or lenvatinib 10–18 mg/m2) in comparison to pembrolizumab monotherapy, in 36 patients with metastatic STS. The ORR, median PFS, and median OS had no significant difference between the three different treatment groups. Leiomyosarcoma had a low response rate to pembrolizumab based treatment, while other histological types including alveolar soft part sarcoma, UPS, extraskeletal chondrosarcoma, and angiosarcoma had a better response rate. There was no association between treatment efficacy and PD-L1 expression but combined treatment (pembrolizumab plus chemotherapy or pembrolizumab plus targeted therapy) could increase the risk for developing severe adverse events. Similarly, in a retrospective study of 28 patients with metastatic STS including angiosarcoma, UPS, epithelioid sarcomas, fibrosarcoma, synovial sarcomas, leiomyosarcomas, pleomorphic liposarcoma, and rhabdomyosarcoma, the nanoparticle albumin-bound paclitaxel was combined with sintilimab in patients with metastatic STS (Tian Z et al. in January 2022) (57). The results showed modest activity and fairly tolerable toxicity with no grade 4 adverse events. Interestingly, patients with angiosarcoma demonstrated significantly longer PFS compared to other subtypes, and among the 5 patients with angiosarcoma included in this study 1 experienced CR (the only patient in the study with CR), 2 had PR, 1 had SD, and 1 experienced PD.

#### 4.1.4 Combinations of immune checkpoint inhibitors with targeted therapies

Contrary to other combinations, the combination of checkpoint inhibitors with tyrosine kinase inhibitors has provided promising results. In particular, the combination the immunotherapy with VEGF targeting multikinase inhibitors has been examined in prospective clinical trials. In a multicentre, single-arm, phase 1b/2 clinical trial, of 68 patients the concurrent inhibition of angiogenesis with sunitinib and the inhibition of PD-1/PD-L1 pathway with nivolumab was examined (73). The primary endpoint was to establish the recommended dose for the phase 2 and to evaluate the 6 months PFS rate. The phase 1 of the trial concluded to a recommended dose of sunitinib of 37.5 mg as induction followed by 25 mg per day, combined with nivolumab. Transaminitis and neutropenia were the most common adverse events, in 17.3% and 11.5%, respectively. The 6 months PFS rate was estimated at 48% (95% CI 41% to 55%), the median OS and the ORR, which were the secondary endpoints, were 24 months and 21%, respectively. Overall, this combination of drugs was tolerated fairly well, the side effects were manageable, and nearly half of the patients did not experience progression at 6 months.

A further phase 2, single centre, single arm clinical trial reported by Wilky BA et al. in 2019 (54) explored the role of the combination of a check point inhibitor (pembrolizumab) and a VEGF targeting multikinase inhibitor (axitinib). Thirty-three patients with advanced sarcomas including 12 patients with alveolar soft part sarcoma, 5 with high-grade pleomorphic sarcoma, 4 with uterine leiomyosarcoma, 2 with non-uterine leiomyosarcoma, 2 with dedifferentiated liposarcoma, and 8 with other types of sarcomas were enrolled in the trial. The patients were treated with escalating doses of axitinib (2-10mg) and flat dose of pembrolizumab (200mg IV on day 8 and every 3 weeks). The 3-month PFS (primary endpoint) for all evaluable patients and for patients with alveolar soft part sarcoma specifically, were 65.6% and 72.7%, respectively. The median PFS was 4.7 months, and the median OS was 18.7 months. Regarding the toxicity, the most common grade 3 or 4 adverse events were hypertension (15%), autoimmune toxicities (15%), nausea or vomiting (6%), seizures (6%), while serious adverse events were reported in 7 patients (21%) and included autoimmune colitis, transaminitis, pneumothorax, haemoptysis, seizures, hypertriglyceridemia. These results were suggestive of clinical benefit particularly for alveolar soft part sarcoma cases and manageable toxicity.

In March 2022 the TAPPAS trial a phase 3, multinational, multicentre, open label, parallel group trial reported the efficacy and safety of TRC105 (carotuximab, an IgG1 antibody binding to endoglin) combined with pazopanib compared to treatment with pazopanib alone in patients with advanced angiosarcoma (89). However, the results were disappointing whilst the PFS (primary endpoint) was not improved with treatment with carotuximab combined with pazopanib compared to pazopanib alone.

In addition, a pilot trial (NCT03282344) which investigated the use of bempegaldesleukin (0.006 mg/kg), a CD122-preferential interleukin-2 pathway agonist, with nivolumab (360 mg/kg every three weeks) in patients with high-grade sarcoma was published in June 2022 by D’Angelo SP demonstrating modest results (45). Eighty-four patients in 9 cohorts were enrolled, based on their histological type including 4 alveolar soft part sarcoma, 10 angiosarcomas, 10 conventional/dedifferentiated chondrosarcomas, 10 leiomyosarcomas, 10 dedifferentiated liposarcomas, 10 osteosarcomas, 6 small blue round cell tumour or synovial sarcoma, 10 UPS or high-grade myxofibrosarcomas, 14 with other types. PR was observed in 1 patient with leiomyosarcoma, 1 with chondrosarcoma, 2 with UPS, 3 patients with angiosarcoma, and 1 with alveolar soft part sarcoma. Overall, ORR (primary endpoint) was highest in patients with angiosarcoma and UPS. The median time to response was 3.7 months and the median duration of response 9.3 months. All patients had at least 1 treatment related adverse event, 30 patients (35%) experienced grade 3-4 toxicity and one death possibly related to therapy was reported.

It is worth mentioning an older retrospective study of Paoluzzi L et al. in 2016 which explored the efficacy of 3mg/kg of nivolumab (IV) every 2 weeks in 28 patients (STS 24, BS 4), 18 of which received concurrently 400-800mg of pazopanib daily (41). Toxicity wise, grade 3-4 adverse events comprised colitis, liver function tests elevations, pneumonitis which occurred in 5 patients. Twenty-four patients were evaluable for response to treatment and the clinical benefit, which corresponded to response and stabilization of disease, was reported in 50% of them. Specifically, 1 patient with dedifferentiated chondrosarcoma on nivolumab, 1 with epithelioid sarcoma and 1 with maxillary osteosarcoma on nivolumab and pazopanib, experienced PR, while 9 patients (including 3 patients with leiomyosarcoma) had SD and 5 of them were on combined treatment. The other 12 patients (including 4 leiomyosarcomas) had progressive disease (PD).Adoptive cellular therapy

### 4.2 Adoptive cellular therapy

Adoptive cellular therapy is a rapidly evolving area in cancer immunotherapy which has the ability to increase the number, specificity, and reactivity of T-cells against malignancies (128). The three major modalities of adoptive cellular therapy include the tumour infiltrating lymphocytes (TILs), genetically engineered T-cell receptors (TCRs) and Chimeric Antigen Receptor (CAR)-T cells. Specifically, TILs therapy requires isolation of TILs from the tumour microenvironment which are expanded ex vivo, away from the immunosuppressive microenvironment, and then are transferred back to the patient; whilst TCRs and CAR-T cells involve expansion of genetically modified T-cells which express T-cell receptors recognizing specific tumour antigens. Importantly, genetically modified TCRs are HLA restricted whilst CAT T-cells identify extracellular antigens independently of HLA presentation. Additionally, lymphodepletion chemotherapy is combined with most forms of adoptive cellular therapy to improve T-cell proliferation and persistence. Multiple factors including inadequate number and function of anti-tumour T cells, and insufficient formation of memory T cells may be involved to resistance mechanisms to immune checkpoint inhibitors, whilst the adoptive cellular therapy has the potential ability to overcome these barriers (128). Several efforts to apply this type of immunotherapy as monotherapy or in combination with immune checkpoint inhibitor or chemotherapy in sarcomas have been made over the last years (**Table S2**, **Supplementary Material**).

#### 4.2.1 TILs therapy combined with chemotherapy or immune checkpoint inhibitors

In order to assess the efficacy of the combination of adjuvant chemotherapy and TILs therapy in patients diagnosed with primary high-grade intramedullary osteosarcoma and had histologically proven poor response to neoadjuvant chemotherapy, Shi J et al., conducted a retrospective analysis which was published in 2020 (105). Of the 80 included in the study, 40 patients comprised group 1 where participants received adjuvant chemotherapy, and another 40 patients comprised group 2 receiving adjuvant chemotherapy and TILs therapy. The neoadjuvant and adjuvant regimens in both groups consisted of high-dose methotrexate, cisplatin and doxorubicin (MAP). It was estimated that the median disease-free survival was 55.5 months and 65.3 months in group 1 and in group 2, respectively. The median OS was 80.4 months in group 1 and 95.8 months in group 2. Additionally, the investigation suggested that a greater number of transfused TILs was an independent prognostic factor for median PFS and OS based on univariate and multivariate analyses. These results indicated that patients with osteosarcoma who responded poorly to neoadjuvant chemotherapy could benefit and have better survival with the combination of adjuvant chemotherapy and TILs therapy.

Another combination approach was presented in two retrospective studies reported in 2020 evaluating the concurrent use of anti-PD1 therapy and TILs therapy in patients with metastatic osteosarcoma. The first retrospective analysis conducted by Zhou X et al. reviewed 60 patients with chemotherapy-resistant metastatic osteosarcoma who were treated with an average of 5x10^9^ cells (range, 3-8x10^9^), TILs per infusion in combination with nivolumab at a dose of 3 mg/kg/cycle. The ORR was estimated at 36.67%, with 2 patients achieving CR and 20 patients PR; the disease control rate was 80% (48 out of 60 patients), the median PFS was 5.75 months, and the median OS was 13.6 months. The most common adverse effects included fever, fatigue, rash, anorexia, leukopenia, and anaemia, and only two patients (3.33%) experienced grade 3 or 4 treatment related adverse events. Importantly, improved PFS and OS were observed in patients with more infusions of TIL and CD8+ TIL, less infusions of CD8+PD1+ TIL and less infusions of CD4+FoxP3+TIL. The results indicated the combination of TILs and anti-PD1 treatment is both safe and effective in patients with refractory metastatic osteosarcoma (106). The second retrospective study by Wang C et al. evaluated the efficacy of anti-PD1 therapy combined with TILs in patients with metastatic osteosarcoma, concluding that this is a potentially promising combination. Thirty patients (group 1) received only nivolumab at 3mg/kg/cycle with a maximum dose of 240mg/cycle and 30 patients (group 2) received combination treatment with an average of TILs per infusion 5.1 × 109 cells (range, 3.2–8.9 × 109). The ORR was estimated at 6.67% and 33.3% for group 1 and group 2, respectively. In group 1 the median PFS was 3.8 months and the median OS was 6.6 months, compared to group 2 in which higher median PFS 5.4 months and OS (15.2 months) was observed. Interestingly, more infusions of TIL number and CD8+ TILs, or fewer infusions of CD8+PD+ TILs or fewer infusions of CD4+FoxP3+ TILs have been associated with prolonged PFS and OS (107).

#### 4.2.2 Genetically modified T-cells

Genetically engineered TCRs and CAR T-cells are directed toward specific tumour antigens (128). Specifically, cancer testis antigens, which are tumour associated antigens, including New York esophageal squamous cell carcinoma 1 (NY-ESO-1) and melanoma-associated antigen (MAGE) are promising targets for sarcoma immunotherapy especially synovial sarcomas and myxoid/round cell liposarcomas (129, 130). Cancer testis antigens are a group of antigens which are normally expressed in testes and are not presented in adult somatic tissues. However, they are also expressed in various malignancies including melanoma, lung cancer and sarcomas, and are likely correlated with tumorigenesis (130). Importantly, NY-ESO-1 has been reported to be expressed in 49-82% of synovial sarcoma and 88-100% in myxoid/round cell liposarcoma, and MAGE is expressed in 45-88% of synovial sarcomas and in 11-68% of myxoid/round cell liposarcoma (130). Overall, NY-ESO-1 and MAGE have been proposed as potential immunotherapy targets for selected subtypes of sarcomas and have been investigated in clinical studies (130).

A phase 1/2 non-randomized, open-label clinical trial was conducted by Ramachandran et al. to illuminate the factors and mechanisms that affect the response and resistance of treatment with NY-ESO-1 SPEAR T-cells, which are genetically modified autologous T cells expressing NY-ESO^c259^, in patients with synovial sarcoma (75). Forty-two patients were enrolled in one of the four cohorts, in which the NY-ESO-1 expression and the lymphodepletion regimen differed between the cohorts. In cohorts 1, 3 and 4 patients expressed the antigen at 2+ or 3+ staining using centralized immunohistochemistry (IHC) in ≥50% of tumour cells, compared to cohort 2 in which patients expressed antigen at 1+ staining by IHC in ≥ 1% of tumour cell, but not 50% or more of cells expressing 2+ or 3+ by IHC. Regarding the lymphodepletion regimen, patients enrolled in cohorts 1 and 2 were treated with fludarabine 30 mg/m^2^/day for 4 days and cyclophosphamide 1800 mg/m^2^/day for 2 days. Participants in cohort 3 were given cyclophosphamide 1800 mg/m^2^/day for 2 days, and patients included in cohort 4 were treated with fludarabine 30 mg/m^2^/day for 3 days and cyclophosphamide 600 mg/m^2^/day for 3 days. The median transduced T-cell dose overall was 2.67 × 10^9^ T-cells. The estimation of the ORR as per RECIST 1.1 was determined as the primary endpoint. Among the 42 patients, CR was reported in 1 patient, PR in 14 patients, SD in 24 patients and PD in 3 patients. Through the performance of this study, it was observed that in order to achieve persistence and efficacy of SPEAR T-cell, high dose fludarabine and cyclophosphamide was required. Additionally, it was suggested that adoptive cellular therapy with gene-modified T cells could be an alternative for non-T-cell inflamed tumours that have poor response to PD-1/PD-L1 inhibitors.

Further to the above, previous clinical studies attempted to evaluate the application of adoptive cell therapy in HLA-*0201 positive patients with synovial sarcoma whose tumours expressed NY-ESO-1 antigen. In 2015 a pilot trial was published, in which 18 HLA-*0201 patients with refractory metastatic synovial cell sarcoma and 20 patients with refractory metastatic melanoma, NY-ESO-1 positive, were treated post lymphodepleting preparative chemotherapy administration with genetically engineered lymphocytes expressing NY-ESO-1 reactive T-cell receptors (76). The lymphodepleting chemotherapy included cyclophosphamide (60 mg/kg/day for 2 days) and fludarabine (25 mg/m^2^/day for 5 days), while the median dose of transduced T cells, which were given 1-3 days after chemotherapy, was 5.5×10^10^ T cells (range from 0.9x10^10^ to 13×10^10^) and additionally interleukin-2 (IL-2) was given at 720,000 IU/kg. Regarding synovial sarcoma, objective clinical response was reported in 11 out 18 patients (61%). The overall 3-year survival rate and 5-year survival rate was estimated to be 38% and 14%, respectively. Of note, the PRs lasted for 3 to 18 months. In comparison, the objective clinical response in the group of patients with melanoma was 55% (11 out of 20 patients), while both the 3-year survival rate and 5-year survival rate were 33%. It was concluded that treatment with autologous T cells transduced with an NY-ESO-1-reactive T-cell receptors could be an effective therapeutic option in patients with synovial sarcoma and melanoma with certain characteristics and resistant to other therapies warranting further investigation.

Another phase 1 clinical trial presented in the 2019 ESMO investigated the use of ADP-A2M4, genetically engineered autologous SPEAR T-cells against MAGE-A4 peptide in HLA-A*02 patients, including participants with inoperable or metastatic synovial sarcoma (79). Ten patients with synovial sarcoma were included and they were treated with median T-cell dose of 9.7x10^9^ (4.49-9.98x10^9^). No dose limiting toxicity was mentioned. Regarding the antitumour activity, 3 confirmed PRs were reported, 1 unconfirmed PR at week 6, 3 SD, 1 PD, and 2 patients were not evaluated. The trial is still ongoing and further data are expected to be reported. Importantly, the ADP-A2M4 (MAGE-A4) was granted Regenerative Medicine Advanced Therapy Designation by FDA in December 2019. Also, SPEARHEAD-1, an open-label phase 2 clinical trial in recruiting status (NCT04044768) is currently investigating the efficacy and safety of ADP-A2M4 SPEAR T-cells in HLA-A*02 positive patients with advanced synovial sarcoma or myxoid/round cell liposarcoma with their tumours being positive for the MAGE-A4 protein (67).

Additionally, in 2015 a clinical trial phase 1/2 conducted by Ahmed N et al. investigated the use of human epidermal growth factor receptor 2 (HER2) - specific CAR T cell in patients with HER2 positive sarcomas (100). Nineteen patients (16 patients with osteosarcoma, 1 with Ewing sarcoma, 1 with primitive neuroectodermal tumour and 1 with desmoplastic small round cell tumour) were enrolled in the study. T cells expressing the HER2-specific chimeric antigen receptor with a CD28.ζ signalling domain were infused. HER2-CAR T cells were given to the participants in escalating doses starting from 1x10^4^/m^2^ to 1x10^8^/m^2^ and no dose limiting toxicity was reported. Among the 19 patients 17 patients were assessable for response to treatment. SD was reported in 4 patients lasting for 12 weeks to 14 months, and 3 of them underwent excision of the tumour with one having ≥90% necrosis indicating the antitumor effect of the treatment. The median OS was 10.3 months with a range from 5.1 to 29.1 months. Interestingly, the persistence of the HER2-CAR T cells was evaluated in 9 patients who were given greater than 1x10^6^/m^2^ HER2-CAR T cells, and it was observed that CARs persisted for at least 6 weeks in 7 patients. A safe dose of HER2-CAR T cells was established through this study. Despite the limited clinical benefit, the possibility of combinations of HER2-CAR T cells with other types of immunotherapies could be further explored.

## 5 Clinical experience with active immunotherapy in sarcomas

### 5.1 Cancer vaccines

Another innovative and challenging immunotherapeutic approach which has been investigated in the treatment of sarcoma is the use of cancer vaccines. Over the past decades, multiple clinical studies on cancer vaccination in sarcoma have been published (**Supplementary Material** **, Table S3**).

Published in 2021 a phase 2, randomized, double-blind, placebo-controlled clinical trial assessed the efficacy of Yeast-Brachyury Vaccine (GI-6301) combined with radiation therapy in patients with locally advanced unresectable chordomas (131). Twenty-four patients were enrolled in the trial 11 patients were assigned in the vaccine arm and 13 patients in the placebo arm, with both arms receiving radiotherapy as well. The results showed no difference in ORR between the two arms and the trial was terminated early. Another phase 1/2 clinical trial conducted by Miwa S et al. evaluating the treatment with autologous tumour lysate pulsed dendritic cells (DCs) in 37 patients with STS or BS showed minimal clinical effectiveness (60). No significant benefit from the treatment with DCs pulsed with autologous tumour lysate in patients diagnosed with sarcoma, was also reported in the phase 1 trial reported by Himoudi N et al. (108), on 16 patients, 13 of which with osteosarcoma. On the other hand, more promising results were reported by Merchant MS et al. in their study on the role of adjuvant immunotherapy with autologous lymphocytes, tumour lysate/keyhole limpet hemocyanin –pulsed DC vaccinations in patients with metastatic or recurrent Ewing sarcoma and rhabdomyosarcoma since these subtypes demonstrated improved outcome compared to previous published studies in this population (80, 132, 133). Forty-three patients (aged <35 at the initial diagnosis) with Ewing sarcoma, rhabdomyosarcoma, desmoplastic small round cell, synovial sarcoma, or undifferentiated sarcoma were enrolled, and 29 finally received immunotherapy with DC-based vaccine ± recombinant human IL7 after the administration of standard chemotherapy regimens. The toxicity of the immunotherapy was tolerable. Significant variability was observed among the different histological subtypes with the 5-year OS being 63% and the PFS 40% in patients with Ewing/rhabdomyosarcoma compared to other sarcomas for which both 5-year OS and PFS were 0%. Therefore, the results suggested that improvement in terms of survival may occur in some histological types of sarcomas with DC-based vaccines (80).

Furthermore, a multicentre, open label, phase 1b clinical trial conducted by Somaiah N et al. evaluated the safety, tolerability and immunogenicity of CMB305, a Lentiviral-Based prime-boost vaccine aimed to produce an anti-ESO-1 immune response, in patients diagnosed with NY-ESO-1 positive locally advanced, or relapsed or metastatic solid malignancies (70). Among the 79 patients who were enrolled in the study, 45 (57%) had progressive disease at study entry, and 64 were diagnosed with sarcomas including myxoid/round cell liposarcoma and synovial sarcoma. There was a 3 + 3 dose-escalation design which was followed by an expansion with CMB305 given as a single regimen or in combination with oral metronomic cyclophosphamide or intratumoral injections of glucopyranosyl lipid A. Toxicity was tolerable with the most common adverse events being fatigue, nausea, and injection-site pain. The estimated disease control rate (defined as immure-related CR, immure-related PR, or immure-related SD, and confirmed by a consecutive assessment at least 4 weeks after first documentation) for sarcoma patients was 61.9% and the OS was 26.2 months. Specifically, the immure-related SD was 61.9% and the immune-related PD was 33.3% for all STS, whilst 4.8% of the tumours were not assessed. Overall, this treatment approach was safe with promising effects indicating further investigation may be worth performing.

Promising results in Ewing’s sarcoma were reported in a previous prospective, non-randomized study published in 2016, in which 16 patients received the Vigil vaccine (GMCSF/bi-shRNA^furin^ DNA-transfected autologous tumour immunotherapy) (112). No significant toxicity was reported and the 1-year survival was 73% and 23% in Vigil-treated patients and non-Vigil treated patients, respectively. A 17.2-month difference in OS between the two groups, was also observed. These results suggested further investigation of Vigil in patients diagnosed with advanced Ewing’s sarcoma could be pursued.

#### 5.1.1 Combinations of oncolytic virus with immune checkpoint inhibitors

Beyond common combinations, one that has nevertheless been tested is the combination of immunotherapy with an oncolytic virus. Oncolytic viruses could be classified into two major categories based on their development, the natural viruses and the genetically modified virus strains (134). Oncolytic viruses have the ability to selectively infect and kill cancer cells whist the normal cells are less susceptible to infection (135). Oncolytic viruses demonstrate multi-mechanistic anti-tumour effects which could be both direct and indirect, and include selective virus replication within targeted cells leading to cytolytic effects, activation of systemic anti-tumour immunity and recruitment of activated immune cells in the tumour microenvironment, and also exert effects of cell death pathways on cancer cells (infected and uninfected) and associated endothelial cell in the tumour-related vasculature eliminating angiogenesis (135). However, mechanisms of oncolytic viruses may vary among different viruses and cancer cell types (135). Several limitations and challenges of the successful function of oncolytic viruses have been descripted (135). Some factors that contribute to the limitation of oncolytic viruses activities and functions include the unknown host anti-viral pathways that restrict oncolytic viruses activity and spread, the selection of the optimal oncolytic virus candidate, the delivery of oncolytic viruses (intratumoral injections are required), the immunosuppressive environment where the oncolytic viruses must function, and the adaptive immune responses that reduce the viral functions (135).

The combination of oncolytic viruses with immune checkpoint inhibitors has been tested in several malignancies in an effort to overcome the limited effectiveness of immune checkpoint inhibitors which is reported in immunologically “cold” tumours (135). Talimogene laherparepvec (T-VEC), an oncolytic immunotherapy developed from a modified human herpes simplex virus type 1, combined with pembrolizumab has been investigated in sarcomas (63). T-VEC has been designed to self-replicate within tumour cells causing their lysis which leads to tumour antigen realise, and regional and systemic antitumour immunity promotion (63). Importantly, T-VEC was approved by FDA for the treatment of melanoma (136).

An open-label, single institution, phase 2 clinical trial assessed the anti-tumour activity of Talimogene laherparepvec (T-VEC) and pembrolizumab in patients diagnosed with locally advanced or metastatic sarcomas (63). The study included 5 patients with leiomyosarcoma, 3 with angiosarcomas, 2 with UPS, 3 with undifferentiated or unclassified sarcoma, and 7 with other histologic subtypes. Both pembrolizumab and T-VEC were given on day 1 of a 21-day cycle. The pembrolizumab was administered IV at a dose of 200mg, while the T-VEC was injected into palpable tumour site/sites with the first dose determined at ≤4 mL × 10^6^ PFU/mL, and the second and subsequent doses at ≤4 mL × 10^8^ PFU/mL. The primary endpoint was met with ORR 30% at 24 weeks. No CR was reported, but 7 patients (35%) experienced PR, 7 patients (35%) had SD, and 6 patients (30%) had PD. The PRs were observed in 5 different histological types and the median duration of response, was 56.1 weeks (range, 49.4-87.0 weeks). Regarding toxicity, 20% (4 patients) experienced grade 3 treatment-related adverse events, and there was no grade 4 adverse events or treatment related deaths. Eleven patients’ samples were tested for PD-L1 expression and TIL characterization before and after treatment. Six patients (55%) turned from PD-L1 negative pre-treatment to PD-L1 positive post-treatment. Only 1 patient was PD-L1 positive before treatment but 4 patients had tested positive after treatment. Also, for the 13 patients with refractory disease, PD-L1 expression was negative pre- treatment but 5 had PD-L1 expression post-treatment. Regarding the TIL score, it was higher in patients who responded to treatment compared to those who did not. In addition, patients who responded to therapy had aggregates of CD3^+^/CD8^+^ TILs at the infiltrating tumour edge in their samples before treatment was applied, and also increased number of CD3^+^/CD8^+^ TILs after treatment. In comparison, in the non-responders’ group both samples pre- and post-therapy had minimal CD3^+^/CD8^+^ TILs infiltration in the tumour, and lack of aggregates TILs at the infiltrating edge of the tumour. Overall, anti-tumour activity has been observed in different types of sarcomas with tolerable toxicity and further investigation of therapy with T-VEC and pembrolizumab in certain histological types of sarcomas is being pursued.

### 5.2 Cytokines

The use of cytokines has also been explored in sarcoma patients. A phase 0 clinical trial published in 2019 conducted by Zhang S et at. investigated the possibility of turning a “cold”, immunosuppresive tumour microenvironment into a “hot” immunosupportive one and thus, enhance additional immunotherapy to act (72). The trial included 8 patients diagnosed with synovial sarcoma or myxoid/round cell liposarcoma, which are sarcoma subtypes considered to have a cold tumour microenvironment. The patients were administered 2-4 weekly injections of IFNγ 100mcg/m^2^ subcutaneously and biopsies were taken before and after the treatment. Tumour microenvironment changes due to IFNγ were observed, and in particular T-cell infiltration and tumour-surface MHC-I expression was reported. Additionally, PD-L1 expression in tumour-infiltrating myeloid cells and tumour cells in certain occasions was increased. This phase 0 trial indicated that IFNγ can convert a “cold” into a “hot” tumour in patients with synovial sarcoma and myxoid/round cell liposarcoma, and the combination with anti-PD1 treatment may produce some advantages in the treatment of this patients.

Earlier, in 2017 Meazza C et al. reported the results of a prospective study which included 35 patients under the age of 18 diagnosed with metastatic osteosarcoma and treated with chemotherapy plus IL-2 (110). Between 1995 and 2010 patients were treated with high dose methotrexate, doxorubicin, cisplatin, ifosfamide, IL-2, LAK (lymphokine-activated killer) reinfusion and surgery. Specifically, 32 participants had their primary tumour excised, 25 underwent lung metastasectomy, and 27 patients were given IL-2 and LAK reinfusion. The determined dose of IL-2 was 9 × 10^6^ IU/sqm/day. The estimated 3-year event-free survival rate was 34.3% and the 5-year EFS was 28.6%. Also, the 3-year OS rate was 45.0% and the 5-year OS was 37.1%. Twenty-four patients experienced a progression or relapse with a median of 10 months after their diagnosis. Twenty-three patients died within 18 months (median) after the diagnosis. Of note, 11 patients remained alive at the time of analysis and importantly all of them underwent surgical excision of both primary tumour and lung metastatic disease, except one who had CR of lung disease. Conclusively, the potential role of IL-2 and LAK/NK cells activation was suggested through the study but further studies are needed to validate the results. Several other clinical studies since the early 1990s presented in **Table S4** (**Supplementary Material)** included patients with BS or STS who were treated with cytokines combined with chemotherapy and/or radiotherapy and/or surgical interventions.

## 6 Potential predictive biomarkers of response to immunotherapy

The rarity and heterogeneity of sarcomas pose challenges in the development of new effective therapeutic strategies but also in the identification of biomarkers that can predict the response of sarcomas to certain therapies. A number of potential biomarkers of response to immunotherapy in sarcoma have been investigated including microsatellite instability (MSI), mismatch repair deficiency (dMMR), tumour mutation burden (TMB), PD-L1 expression, infiltration of TILs, B cell-related gene signature and presence of intratumoral tertiary lymphoid structures (TLSs) (137, 138).

FDA approved mismatch repair deficiency and microsatellite instability as a biomarker for immune response to pembrolizumab, for solid tumours independently of histological type or site of malignancy (137, 139). The use of dMMR/MSI as a predictive biomarker of immunotherapy has great clinical value in some cancers, especially in colorectal cancer (137, 140). The potential of dMMR/MSI as predictive biomarker has also been assessed in sarcomas but the results are conflicting (137). Through their study, Campanella NC et al. observed, the absence of MSI in 71 patients with STS who were examined (141). Compatible with this result were several other studies including patients with either BS or STS (142–144). Conversely, other studies have identified dMMR/MSI in sarcoma patients (145–147) but further studies are required to delineate the role of this marker in sarcomas.

TMB has an established role in the treatment of several cancers (137). For example, it correlates with the response to nivolumab in patients with non-small cell lung cancer, and it has a predictive value in melanoma patients and their response to immunotherapy (148, 149). However, its role in sarcomas is not thoroughly investigated and its clinical value has not been established yet. Cote GM et al. reported a retrospective analysis of 133 tumour samples of sarcoma patients following next generation sequencing. Low or intermediate TMB levels were observed in almost all samples, except in two samples (1 UPS, 1 high‐grade STS with leiomyosarcoma features) (150). The role of TMB has also been investigated through the Angiosarcoma Project, where it was quantified in 47 angiosarcoma samples (151); angiosarcoma being an aggressive sarcoma subtype with very limited therapeutic options at present (152). Interestingly, the median mutational burden was estimated at 3.3 mutations per megabase in full cohort, but it appeared to be much higher in head/neck/face/scalp angiosarcoma samples (20.7 mutations per megabase) compared to non-head/neck/face/scalp angiosarcomas (2.8 mutations per megabase). Of note, 3 patients with head/neck/face/scalp angiosarcoma and high TMB were treated with off-label pembrolizumab and 2 of them experienced durable response to this treatment, while the third patient stopped the treatment after the first dose due to toxicity. In contrast, 3 patients with non-head/neck/face/scalp angiosarcomas, low TMB, and no dominant mutational signature of ultraviolet light, derived no clinical benefit from PD1 checkpoint inhibitors. These results suggest that TMB may have a predictive role in patients with head/neck/face/scalp angiosarcomas who receive immune check point inhibitors.

In clinical practice an important predictive biomarker of immunotherapy in a variety of malignancies is the expression of the PD-L1 (137, 153, 154). Inevitably, the role of PD-L1 expression has been studied in sarcomas as well. However, among clinical studies there is a diversity regarding the expression of PD-L1 among different histological types of sarcomas and the role of PD-L1 as a predictive biomarker is not clear (137). A retrospective analysis by Starzer AM et al. showed no association between PD-L1 expression and response to immunotherapy, which is also supported by other clinical studies showing no correlation between PD-L1 and treatment efficacy (37, 53). Interestingly, in the SARC028 trial, only 3 cases had PD-L1 expression among the 70 tissue samples that were examined. All three patients were diagnosed with UPS and 2 of them had response to pembrolizumab (47). Currently, there is no established role of PD-L1 expression as a predictive biomarker in sarcomas (137).

Tumour immune microenvironment is another marker which may have a potential role in the prediction of response to immune checkpoint inhibitors (137, 155). TILs, which are part of tumour microenvironment, are considered to be a prognostic factor in multiple malignancies, and the density of TILs in the tumour microenvironment has been correlated with clinical advantages from immunotherapy with immune checkpoint inhibitors (137). The evidence related to the use of TILs as a predictive biomarker of immune checkpoint blockage in patients diagnosed with sarcomas is not sufficient yet, so further investigation is required (137).

The presence of intratumoral tertiary lymphoid structures, which are aggregates consisted of B cell-rich areas, T cells and follicular dendritic cells, has been proposed as a predictive biomarker for the treatment with immune checkpoint inhibitors in STS (50, 138). Petitprez F et al. investigated the gene expression profiles in more than 600 STS tumours, and established an immune-based classification in sarcomas according to the composition of the tumour microenvironment (138). Particularly, five sarcoma immune class (SIC) phenotypes were identified comprise of SIC-A and SIC-B which were characterised as low immune activity groups, SIC-D and SIC-E which were high immune activity groups, and SIC-C which was high vascularized group. SIC-E was characterised by high expression of B cell-related gene signature, which was predictive of survival irrespectively of the infiltration level of CD8^+^T cell, and by the presence of intratumoral TLSs based on immunohistochemistry analysis. Additionally, investigators proceeded to a retrospective analysis of samples taken from 47 patients who were included in the SARC028 trial and were further classified in one of the five SIC phenotypes. Analysis showed that those patients who were categorised as SIC-E group had improved survival and higher response rate to pembrolizumab compared to other SIC groups indicating that the presence of TLSs in STS might represent a predictive biomarker (138). Based on these results, the PEMBROSARC trial introduced the biomarker-driven cohort which included TLS-positive STS treated with cyclophosphamide and pembrolizumab with the results of this cohort further supporting that presence of intratumoral TLSs could predict response to immune checkpoint inhibitors in STS (50).

## 7 Discussion

The high rate of relapse in high-grade localized sarcomas, the poor prognosis in metastatic disease and the limited range of effective therapies, create an urgent need for new, effective therapeutic strategies to be developed. Different types of immunotherapy approaches have been tested in patients with sarcoma through multiple clinical trials as presented in this review.

It appears that different histological types of sarcomas may respond differently to immunotherapy, but the results of clinical trials are not always in agreement with each other. UPS for example, may have a meaningful response to immune checkpoint inhibitors. This is supported by two important clinical trials the SARC028 and Alliance A091401, in which patients were treated with pembrolizumab (SARC028) and with nivolumab versus nivolumab plus ipilimumab (Alliance A091401 trial) respectively (38, 47). Similar results were reported also by large retrospective studies (40, 53) and a systematic review and meta-analysis (156). On the other hand, limited efficacy has been shown in a phase 2 clinical trial reported by Toulmonde M et al. where metronomic cyclophosphamide and pembrolizumab were used in patients with UPS (49). Conflicting results have also been reported for leiomyosarcomas. The clinical activity and response to single immune checkpoint inhibitor appeared poor in studies including the SARC028 (47), a phase 2 clinical trial conducted by Toulmonde M (49), a retrospective study reported by Liu J et al. (53), and a phase 2 trial conducted by Ben-Ami E et al. which was terminated early due to a lack of benefit of nivolumab in patients with advanced uterine leiomyosarcoma (42). However, the combination of nivolumab and ipilimumab provided more promising results in leiomyosarcomas, in the Alliance A091401 (38) and a retrospective analysis by Monga V et al. (40).

In other sarcoma subtypes, results supporting the benefit or lack of benefit of immunotherapy, are more consistent. In alveolar soft tissue part sarcomas, for example, there is cumulative evidence of response to certain immune checkpoint inhibitors. In a phase 2 clinical trial conducted by Wilky BA et al. in which patients were treated with pembrolizumab and axitinib, the 3-month PFS was 72.7% (54). Also, geptanolimab provided promising results in a phase 2 trial by Shi YK et al. (85). An ongoing phase 2 clinical trial (NCT03141684) has shown encouraging results so far of the use of atezolizumab in patients with alveolar soft part sarcomas (86). Additional evidence regarding the use of immune checkpoint inhibitors, has been provided by a systematic review and a meta-analysis performed by Saerens M et al. (156), as well as the retrospective analysis of data collected from a world-wide registry in which sixty patients with alveolar soft part sarcomas and treated with PD1/PD-L1 in Europe, Australia and USA (157). Conversely, in synovial sarcomas, the role of immune checkpoint inhibitors is limited as reported in several trials (47, 74) but interestingly, the role of adoptive cellular therapy might be promising. A phase 1/2 clinical trial reported by Ramachandran I et al. (75), and also a pilot trial conducted by Robbins PF indicating further investigation (76), provide preliminary evidence that patients with synovial sarcoma and “cold” tumours with minimal response to immune checkpoint inhibitors, may derive benefit from adaptive cellular therapy. With regards to osteosarcoma, single immune checkpoint inhibition is of limited value but the combination of anti-PDL-1 and TILs therapy in metastatic disease, showed safety and effectiveness, in two retrospective studies published in 2020 (106, 107). Also notable, the use of mifamurtide, an innate immunity modulator, has been approved in Europe for patients with non-metastatic osteosarcoma, based on a phase 3 clinical trial in which mifamurtide was combined with conventional chemotherapy and showed better outcomes compared to patients treated with chemotherapy alone (158).

The reasons why different sarcoma subtypes, may respond differently to immunotherapy remain unclear. What is clear however, is that with the exception of SARC028, single agent immunotherapy treatment responses in unselected sarcoma populations have been disappointing, raising the question whether the results of this trial occurred by chance. To this end, within SARC028, a further investigation of the characteristics that might have been related with the response to pembrolizumab in UPS patients, was performed. PD-L1 expression was noted in 3 patients with UPS, one having a CR and one having a PR. The PD-L1 expression correlating with infiltration of T cells may suggest that UPS is indeed an “inflamed” malignancy leading to response to PD-1 inhibitors. Other studies also support that UPS is a “hot” tumour with high tumour infiltrating lymphocytes, making patients possible candidates for treatment with immune check point inhibitors (159). On the other hand, responses have been reported in patients with UPS who have no PD-L1 expression, which indicates that the actual role and importance of PD-L1 expression has not been fully clarified yet (47).

The expression of PD-L1 in STS has been reported in different studies over the last decade but the results have been variable. In some studies, the percentage of tumour cells expressing PD-L1 appears to be as high as 58% and 59% (159, 160), whilst in others it is reported to be as low as 6.6% and 12% (161, 162). Admittedly, the number of sarcoma samples tested in each study is different, the assay used may be different and the sarcoma subtypes included in each study may also be different. In the study by Kim et al. (160) of 105 cases PD-L1 expression was significantly associated with higher clinical stage, presence of distant metastasis, higher histological grade, poor differentiation of tumour, and tumour necrosis. In the multivariate analysis, PD-L1 was reported as an independent prognostic indicator of OS. In contrast, in the study by D’Angelo (162) of 50 STS cases, there was no association between clinical features, OS and PD-L1 expression in tumour. Regarding specific subtypes, in the study by Pollack et al. (159) UPS were found to have higher levels of PD-L1 (P≤.001) and PD-1 (P≤.05) expression, significantly more than other subtypes including liposarcoma or synovial sarcoma, which had the lowest (P≤.05).

Regarding the use of active immunotherapy (vaccines, cytokines) although explored in multiple types of sarcomas, there has been diversity in efficacy among clinical studies (60, 70, 80, 108, 112, 131). There might be merit in further investigating the role of active immunotherapy in Ewing sarcoma based on encouraging results on a DC-based vaccination (80) and the Vigil vaccine (112). Regarding treatment with cytokines, considered a promising treatment back in the 1990s, the results of several clinical trials showed that IFNγ as a single immunotherapeutic agent is not effective (72, 163). Currently, the interest is directed to further explore changes in tumour microenvironment that can be induced by IFNγ and also to investigate the combination of IFNγ with other types of immunotherapies and especially with anti-PD1 treatment (72). Some sarcoma subtypes such as synovial sarcoma and myxoid/round cell liposarcoma characterized as “cold” tumours without high expression of MHC-I and T-cells infiltration, may turn into hot tumours by IFNγ and thus may benefit from combination treatment with IFNγ and other immunotherapy strategies. The phase 2 clinical trial NCT03063632 for example, investigating the combination of IFNγ and pembrolizumab, included patients with synovial sarcoma as one of its cohorts.

To conclude, sarcomas constitute a rare group of malignancies characterized by extensive heterogeneity. Metastatic disease is associated with poor prognosis and new therapeutic avenues are intensively explored. Immunotherapy, being a rapidly expanding field in oncology, has emerged as a potential therapeutic strategy in sarcomas although its clinical activity has not been impressive to date. There are several challenges around the use of immunotherapy in sarcomas including the great heterogeneity of mesenchymal origin malignancies, the absence of antigens that could be potential targets for vaccines or adoptive cellular therapy or antibodies, and the lack of sufficient understanding of tumour microenvironment characteristics. There is also an unfulfilled need for the identification of potential predictive biomarkers of immunotherapy in sarcomas. Whilst results of clinical trials on some sarcoma subtypes, may provide a glimpse of promising data, further studies are essential to delineate the role of immunotherapy among the different subtypes of sarcomas, particularly in combination therapy.

## Author contributions

AC and TS conceptualized the study and all authors were involved in the design of the review. Both PP and MP contributed to the collection of relevant literature and data. PP wrote the first draft of the manuscript. SA and RJ contributed in the review and editing of the manuscript. Both TS and AC reviewed, revised and edited the final version of the manuscript. All authors read and approved the final manuscript. All authors contributed to the article and approved the submitted version.
