# Supplementary material for: Exploring the landscape of immunotherapy approaches in sarcomas
Source: Front Oncol. 2023 Jan 9;12:1069963. doi: 10.3389/fonc.2022.1069963 (PMC9853527; doi:10.3389/fonc.2022.1069963)
Supplement: Supplementary file 1 [file DataSheet_1.pdf]

Supplementary Material

Table S1: Clinical experience of immunomodulating antibodies in sarcoma patients

No	Authors	Year	Type of study	Drug/Agent used	Treatment course	Number of patients	Type of sarcoma	Outcome
1	Italiano A et al. (1)	2022	Phase 2	Pembrolizumab & Metronomic cyclophosphamide	-Pembrolizumab: IV 200 mg every 3 weeks (day 8 of a 21-day cycle)-Cyclophosphamide: orally 50 mg bd 1 week on / 1 week off	35	Intratumoral tertiary lymphoid structures positive STS (12 well-differentiated/dedifferentiated liposarcomas, 4 leiomyosarcomas, 6 undifferentiated pleomorphic sarcomas, 10 other types)	-30 evaluable for efficacy<br>-6-month non-progression rate: 40% (95% CI, 22.7-59.4)<br>-ORR was 30% (95% CI, 14.7–49.4)<br>-PR: 9 patients<br>-SD: 10 patients<br>-PD: 10 patients<br>-most frequent toxicities: grade 1 or 2 fatigue, nausea, dysthyroidism, diarrhoea & anaemia
2	Gordon EM et al. (2)	2022	Phase 2	Ipilimumab, Nivolumab & Trabectedin	-Ipilimumab: 1 mg/kg IV q 12 weeks<br>-Nivolumab: 3 mg/kg IV q 2 weeks<br>-Trabectedin 1.2 mg/m^2 IV q 3 weeks	99	Locally advanced or unresectable or metastatic STS	-88 evaluable for efficacy<br>-ORR: 21.6%<br>-DCR: 87.5%<br>-Median OS: 14 months<br>-Median PFS: 7 months
3	Wagner MJ et al. (3)	2022	Phase 1/2	Avelumab & Trabectedin	-Avelumab: 800mg<br>-Trabectedin: 1, 1.2, and 1.5 mg/m^2	33	24 Leiomyosarcomas (6 uterine and 18 non uterine), 11 Liposarcomas	-Recommended phase 2 dose for trabectedin: 1.0 mg/m^2<br>-PR: 3 patients (13%)<br>-SD: 10 patients (43%)<br>-6-month PFS: 52%<br>-median PFS: 8.3 months
4	Toulmonde M. et al. (4, 5)	2022	Phase 1b	Trabectedin & Durvalumab	-3+3 design<br>-Maximum tolerated dose: 1.2 mg/m^2 for trabectedin and 1120 mg/m^2 for durvalumab	16	Unresectable or metastatic STS	-Grade 3/4 adverse events: 8 patients (50%)<br>-Grade 5 adverse events: 2 patients (multi-organ failure & febrile aplasia)<br>-ORR: 7.1% (1 PR)<br>-6-month PFR: 28.6%

5	Salkeni MA et al. (6)	2022	Phase 2	Atezolizumab	1200mg once every 21 days	9	Unresectable conventional chondrosarcoma (grade 2 & 3)	-No objective responses<br>-SD: 33% (three patients)
6	D'Angelo SP et al. (7)	2022	Pilot study	Bempegaldesleukin & nivolumab	-bempegaldesleukin: 0.006 mg/kg<br>-nivolumab: 360 mg/kg every 3 weeks	84	High-grade sarcomas (4 alveolar soft part sarcoma, 10 angiosarcomas, 10 conventional/dedifferentiated chondrosarcoma, 10 leiomyosarcoma, 10 dedifferentiated liposarcoma, 10 osteosarcoma, 6 small blue round cell tumour or synovial sarcoma, 10 undifferentiated pleomorphic sarcoma or high-grade myxofibrosarcoma, 14 with other types	-77 patients evaluable for efficacy:<br>(1) alveolar soft part sarcoma: PR (1/4), median PFS (2.6 months), median OS (NE)<br>(2) angiosarcomas: PR (3/8), median PFS (7.3 months), median OS (NE)<br>(3) conventional/dedifferentiated chondrosarcoma: PR (1/10), median PFS (1.8 months), median OS (5.1 months)<br>(4) leiomyosarcoma: PR (1/10), median PFS (1.8 months), median OS (6.9 months)<br>(5) dedifferentiated liposarcoma: PR (0/10), median PFS (3.9 months), median OS (21.7 months)<br>(6) osteosarcoma: PR (0/10), median PFS (2.0 months), median OS (6.3 months)<br>(7) small blue round cell tumour or synovial sarcoma: PR (0/5), median PFS (2 months), median OS (5.9 months)<br>(8) undifferentiated pleomorphic sarcoma or high-grade myxofibrosarcoma: PR (2/10), median PFS (2.4 months), median OS (9.2 months)<br>(9) other types: PR (1/10), median PFS (2.1 months), median OS (12.0 months)
7	Somaiah N et al. (8)	2022	Phase 2	Durvalumab & Tremelimumab	-Durvalumab: 1500mg<br>-Tremelimumab: 75mg<br>-Durvalumab & Tremelimumab every 4	62	Advanced metastatic sarcomas (liposarcoma, leiomyosarcoma, angiosarcoma,	-57 received treatment and were evaluable for efficacy

					weeks for 4 cycles followed by durvalumab alone every 4 weeks for up to 12 months		undifferentiated pleomorphic sarcoma, synovial sarcoma, osteosarcoma, alveolar soft part sarcoma, chordoma, and other sarcomas)	-12 weeks PFS: 49% (80% for patients with alveolar soft part sarcoma, 17% for patients with liposarcoma)<br>-Median PFS: 2.8 months (95%CI 1.8-6.4)<br>-Median OS: 21.6 months (95% CI 12.3-30.9)
8	Adnan N et al. (9)	2022	Phase 2	Metronomic gemcitabine, doxorubicin, docetaxel & nivolumab	On Day 1 and Day 8:<br>-Gemcitabine: 600 mg/m^2, max:1000 mg<br>-Doxorubicin: 18 mg/ m^2, max: 32 mg<br>-Docetaxel: 25 mg/m^2, max:42 mg<br>On day 1 only:<br>-Nivolumab: 240 mg	43	15 leiomyosarcomas, 4 pleomorphic sarcomas, 4 synovial sarcoma, 3 liposarcomas, 3 osteosarcomas, 10 other sarcomas	-median PFS: >4.6 months<br>-median OS: 6.2 months
9	Cousin S et al. (10)	2022	Phase 2	Avelumab & Regorafenib	-Avelumab: 10 mg/kg every 2 weeks<br>-Regorafenib: 160 mg QD 3weeks/4	49	Advanced STS (22 leiomyosarcoma, 9 synovial sarcoma, 4 liposarcoma, 4 undifferentiated pleomorphic sarcoma and 10 others subtypes)	-PR: 4 patients (9.3%)<br>-SD: 17 patients (39.5%)<br>-PD: 22 patients (51.2%)<br>-median PFS: 1.8 months (95% 1.7-3.5)<br>-median OS: 15.1 months (95% 7.2-NA)
10	Jones RL et al. (11)	2022	Phase 3	TRC105 (carotuximab) & pazopanib vs pazopanib alone (randomized 1:1)	-Arm A: oral pazopanib 800 mg/day<br>-Arm B: IV carotuximab 10mg/kg weekly plus oral pazopanib 800 mg/day	123	Advanced angiosarcoma	-114 evaluable patients (53 in the pazopanib arm, 61 in the carotuximab & pazopanib arm)<br>-PFS (primary end point) was not reached: median 4.2 months (combination arm), and median 4.3 months for pazopanib.
11	Tian Z et al. (12)	2022	Retrospective study	Nab-paclitaxel & Sintilimab	-Nab-paclitaxel: 300 mg/m^2<br>-Sintilimab: 200mg IV	28	Metastatic STS (5 angiosarcoma, 7 undifferentiated pleomorphic sarcomas, 5 epithelioid sarcomas, 4 fibrosarcoma, 3 synovial sarcomas, 2 leiomyosarcomas, 1 pleomorphic	-ORR: 25%<br>-DCR: 50%<br>-Median PFS: 2.25 months<br>-No grade 4 adverse events

							liposarcoma, 1 rhabdomyosarcoma)	
12	Livingston MB et al. (13)	2021	Phase 2	Pembrolizumab & Doxorubicin	-Pembrolizumab: 200 mg IV every 21 days<br>-Doxorubicin: 60 mg/m^2 cycle 1 with escalation to 75 mg/m^2 on cycle 2	30	Anthracycline-naïve STS (7 liposarcomas, 10 leiomyosarcomas, 1 synovial sarcoma, 4 undifferentiated pleomorphic sarcomas, 2 angiosarcomas, 1 rhabdomyosarcoma, 1 epithelioid angiosarcoma, 1 Fibromyxoid sarcoma/sclerosing epithelioid fibrosarcoma, 1 Malignant fibrous histiocytoma, 1 Spindle-cell solitary fibrous tumor, 1 Extraskeletal osteosarcoma	-ORR: 36.7%.<br>-CR: 1 patient (3.3%)<br>-PR: 10 patients (33.3%)<br>-SD: 13 patients (43.3%)<br>-median PFS: 5.7 months (95% CI 4.1-8.9)<br>-median OS: 17 months (95% CI 9.9-NR).
13	Wagner MJ et al. (14)	2021	Phase 2	Ipilimumab & Nivolumab	-Ipilimumab: IV 1mg/kg every 6 weeks<br>-Nivolumab: IV 240mg every 2 weeks	16	Metastatic or unresectable angiosarcoma	-ORR: 25% (4/16)<br>-6-month PFS: 38%
14	Naqash AR et al. (15)	2021	Phase 2	Atezolizumab	Adults: 1200mg of atezolizumab<br>-Paediatric patients: 15mg/kg (1200 mg max)	44	Alveolar soft part sarcoma	-43 evaluable for response<br>-ORR: 37.2%<br>-CR: 1 patient<br>-PR: 15 patients<br>-SD: 25 patients
15	Smrke A. et al. (16)	2021	Phase 1	Gemcitabine & Pembrolizumab	-3+3 design: 800 mg/m^2, 1000 mg/m^2, 1200 mg/m^2 of gemcitabine<br>-Pembrolizumab: 200mg IV on day 1 of 21-day cycle	13	Advanced leiomyosarcoma (n=11) and undifferentiated pleomorphic sarcoma (n=2)	-Maximum tolerated dose was not reached<br>-Recommended dose of gemcitabine: 1200 mg/m^2<br>-16 serious adverse events were reported (fever was the most frequent)<br>-Median PFS: 5.1 months
16	You Y et al. (17)	2021	Retrospective study	ICI ± TKIs	-ICI: PD1/ PDL1 inhibitors	61	Advanced STS (20 leiomyosarcoma, 17 dedifferentiated	- Median PFS:<br>(a) ICI+TKIs: 11.74 months<br>(b) ICI alone: 6.81 months

					-TKIs: anlotinib, pazopanib, regorafenib<br>-ICI alone: 21 patients<br>-ICI & TKIs: 40 patients		liposarcomas, 8 undifferentiated pleomorphic sarcomas, 7 alveolar soft part sarcomas, 7 myxofibrosarcomas, 2 angiosarcoma)	-ORR:<br>(a) ICI+TKIs: 30% (alveolar soft part sarcomas 66.7%, myxofibrosarcomas 42.9%, undifferentiated pleomorphic sarcoma 33.3%)<br>(b) ICI alone: 9.5%
17	Li Y et al. (18)	2021	Retrospective study	Camrelizumab & apatinib	Camrelizumab & apatinib within 5 days of local interventional therapy using transarterial chemoembolization or radiofrequency ablation (RFA)	33	Metastatic STS	-Median PFS: 8.8 months<br>-Median OS: 18.5 months<br>-ORR: 36.4%<br>-DCR: 75.8%
18	Wang J et al. (19)	2021	Retrospective study	Apatinib and/or Camrelizumab	-Apatinib monotherapy: 5 patients<br>-Apatinib & camrelizumab: 4 patients<br>-Carmelizumab: 3 patients	12	Advanced clear cell sarcoma	-Among the 12 patients: 3 PRs, 4 SDs<br>-Apatinib monotherapy: 1 PR, 2 SD<br>-Apatinib & camrelizumab: 1 PR, 2 SD<br>-Carmelizumab: 3 PD (addition of apatinib afterwards → 1 PR, 1 SD were observed)
19	Doshi A et al. (20)	2021	Retrospective study	Cryoablation combined with ipilimumab & nivolumab	Ipilimumab (1mg/kg) & Nivolumab (3mg/kg) every 3 weeks for 4 (max) induction cycles followed by nivolumab 3mg/kg every 2 weeks or 6mg/kg every 4 weeks.	16	Metastatic STS (leiomyosarcoma, liposarcomas, myxofibrosarcomas, malignant peripheral nerve sheath tumour, synovial sarcoma, Sarcoma NOS, Hemangiopericytoma)	-Clinical benefit: 7 patients (1 CR, 1 PR, 5 SD, 9 PD)<br>-Median OS: 14.1 months<br>-Median PFS: 2.3 months
20	Scheinberg T et al. (21)	2021	Retrospective study	Pembrolizumab	IV pembrolizumab 2 mg/kg every three weeks	18	Advanced bone sarcoma & STS<br>(6 Ewing sarcomas, 4 osteosarcomas, 3 synovial sarcomas, 3 alveolar soft part sarcoma, 1 embryonal rhabdomyosarcoma, 1 clear cell sarcoma)	-14 evaluable patients<br>-CR: 1 patient (Ewing sarcoma)<br>-PR: 1 patient (alveolar soft part sarcoma)<br>-SD: 1 patient (alveolar soft part sarcoma)<br>-PD: 11 patients

21	Starzer AM et al. (22)	2021	Retrospective study	Anti-DP-1 therapy:<br>-pembrolizumab<br>-nivolumab	-Pembrolizumab: 30 patients<br>-Nivolumab: 5 patients	35	Recurrent sarcomas: 27 STS, 8 osteosarcomas	-Objective response rate to anti-PD-1: 22.9%<br>-Clinical benefit rate of 45.7%<br>-CR: 3/35<br>-PR: 5/35<br>-SD: 8/35<br>-Median PFS: 3.0 months<br>-Median overall survival: 16.5 months
22	Liu J et al. (23)	2021	Retrospective study	Pembrolizumab; as monotherapy (group A) or combined with ChT (group B) or combined with targeted therapy (group C)	-Group A (6 patients): pembrolizumab injections 2 mg/kg for 21 days per cycle<br>-Group B (16 patients): pembrolizumab + ChT (AIM, AD, high dose ifosfamide, gemcitabine with docetaxel or dacarbazine)<br>-Group C (16 patients): pembrolizumab + targeted therapy (anlotinib 12 mg/day or pazopanib 400–600 mg/day or lenvatinib 10–18 mg/ m^2)	38	Advanced soft tissue Sarcoma (9 leiomyosarcomas, 4 undifferentiated pleomorphic sarcomas, 5 alveolar soft part sarcomas, 2 synovial sarcomas, 4 pleomorphic rhabdomyosarcoma, 4 clear cell sarcomas, 4 dedifferentiated liposarcomas, 1 epithelioid sarcoma, 1 malignant peripheral nerve sheath tumour, 1 angiosarcoma, 1 carcinosarcoma, 1 exoskeletal chondrosarcoma, 1 desmoplastic small round cell tumour)	-36 evaluable patients<br>-ORR: 19.4% (7/36)<br>-Median PFS: 2.9 months<br>-Median OS: 12.0 months
23	Naing A et al. (24)	2021	Phase 1/2	CX-072 (pacmilimab)	-IV administration every 14 days.<br>-Dose-escalation phase: 0.03, 0.1, 0.3, 1, 3, 10, and 30mg/ kg of pacmilimab.	22	-Uterine sarcoma (2 patients in dose-escalation phase)<br>-Undifferentiated pleomorphic sarcoma (20 patients in expansion phase)	-Recommended phase 2 dose: 10mg/kg.<br>-Objective responses: 1 patient with undifferentiated pleomorphic sarcoma had PR<br>-SD: 4 patients with undifferentiated pleomorphic sarcomas<br>-PD: 12 patients with undifferentiated pleomorphic sarcomas<br>-Early discontinuation: 3 patients with undifferentiated pleomorphic sarcomas

24	Pollack SM (25)	2020	Phase 1/2	Doxorubicin & Pembrolizumab	-2 dose levels of doxorubicin: 45 and 75 mg/ m^2<br>-Pembrolizumab dose: 200mg IV	37	Advanced, anthracycline-naive sarcomas	-Treatment tolerated well. No dose-limiting toxic effects.<br>-Phase 2 dose: 75 mg/ m^2<br>-ORR for phase 2: 13%<br>-ORR overall: 19%<br>-Median PFS: 8.1 months<br>-Median OS: 27.6 months
25	Martin-Broto J et al. (26)	2020	Phase 1b/2	Nivolumab & Sunitinib	2 dose levels:<br>-level 0: sunitinib 37.5mg daily from day 1 & nivolumab 3mg/kg IV on day 15, then every 2weeks<br>-level -1: sunitinib 37.5mg on the first 14 days (induction) and then 25mg/day & nivolumab (same schedule)	68	Advanced STS (16 patients in phase Ib and 52 in phase II)	-Recommended dose: sunitinib 37.5mg (induction), and then 25mg combined with nivolumab.<br>-Most common grade 3–4 adverse events: transaminitis (17.3%) and neutropenia (11.5%).<br>-6-month progression-free survival rate: 48%<br>-Median OS: 24 months<br>-ORR: 21%
26	Roland CL et al. (27)	2020	Phase 2	-Nivolumab or<br>-Combination of ipilimumab & nivolumab	-Neoadjuvant setting<br>-Concomitant neoadjuvant radiotherapy	25	Surgically resectable retroperitoneal dedifferentiated liposarcoma or extremity/ truncal undifferentiated pleomorphic sarcoma	-24 evaluable patients<br>-Median pathological response:<br>(a)Undifferentiated pleomorphic sarcoma: 95%<br>(b)Dedifferentiated liposarcoma: 22.5%
27	Nathenson MJ et al. (28)	2020	Phase 2	Eribulin & Pembrolizumab	-Eribulin: 1.4mg/m^2(day 1, 8)<br>-Pembrolizumab: 200mg (day 1), every 21 days	19	Metastatic STS: leiomyosarcoma cohort	12 weeks PFS (primary endpoint): 42.1% (did not meet the predefined endpoint)
28	Blay J et al. (29)	2020	Phase 2	Pembrolizumab	-Pembrolizumab: 200mg IV on day 1 of 21-day cycle	80	Rare sarcomas: 24 chordoma, 13 alveolar soft-part sarcoma, 6 desmoplastic small round cell tumour, 6 smarca4-malignant rhabdoid tumour and 31 other types	-PR: 13 patients (16.25%)<br>-SD: 29 patients (36.25%)<br>-1-year PFS rate:<br>(1) chordoma: 35%<br>(2) alveolar soft-part sarcoma: 58%<br>(3) desmoplastic small round cell tumour: 0<br>(4) smarca4-malignant rhabdoid tumour: 62.5%<br>(5) other types: 8%
29	Kawai A et al. (30)	2020	Phase 2	Nivolumab	Nivolumab: 240mg every 2 weeks	25	Unresectable clear cell sarcoma (n=11) and	- Response rate (central review):

							alveolar soft part sarcoma (n=14)	(a)PR: 4% (1 patient with alveolar soft part sarcoma)<br>(b)SD: 60% (15 patients)<br>(c)PD: 32% (8 patients)<br>-Median PFS: 4.9 months<br>-Median OS: 15.8 months
30	Naing A et al. (31)	2020	Phase 2	Pembrolizumab	-IV administration<br>-Dose: 200mg every 21 days	>7	Vascular sarcoma (n=7) + other rare tumour histologies	-Immune-related PR: alveolar soft part sarcoma, epithelioid neoplasm of the dermis.<br>-Immune-related SD (≥4 months): 2 vascular sarcomas, sclerosing epithelioid fibrosarcoma
31	Shi YK et al. (32)	2020	Phase 2	Geptanolimab (GB226)	-IV administration<br>-Dose: 3 mg/kg every 2 weeks	37	Unresectable, recurrent, or metastatic alveolar soft part sarcoma	-Objective response: 14/37 patients (37.8%)<br>-6-month response rate: 91.7%<br>-Median PFS: 6.9 months<br>-Disease control: 32 patients (86.5%)<br>-Grade 3 adverse events: anaemia, hypophysitis, proteinuria (1 patient each)<br>-No grade 4 adverse events
32	Naing A et al. (33)	2020	Phase 1	Spartalizumab	-IV administration<br>-Doses: 1, 3, or 10mg/kg, every 2 weeks, or 3 or 5mg/kg every 4 weeks	16	Sarcoma	-Recommended doses: 400mg every 4 weeks or 300mg every 3 weeks.<br>-No dose-limiting toxicities.<br>-Most common adverse events: fatigue, diarrhoea, pruritus, hypothyroidism, nausea.
33	Zhou M et al. (34)	2020	Retrospective study	Nivolumab & Ipilimumab	IV ipilimumab 1 mg/kg and nivolumab 3 mg/kg every 3 weeks (max 4 cycles) followed by nivolumab monotherapy 3 mg/kg every 2 weeks or 6 mg/kg every 4 weeks.	38	-Unresectable or metastatic STS (9 leiomyosarcomas, 8 sarcomas not otherwise specified, 6 liposarcomas, 5 myxofibrosarcoma, 3 malignant peripheral nerve sheath tumour, 2 solitary fibrous tumour, 1 breast angiosarcoma, 1 fibrosarcoma	-CR: 1 patient (myxofibrosarcoma)<br>-PR: 5 patients (malignant peripheral nerve sheath tumour, solitary fibrous tumour, myxofibrosarcoma, dedifferentiated liposarcomas, sarcoma not otherwise specified)<br>-SD: 7 patients<br>-PD:25 patients<br>-ORR: 15%<br>-Median OS: 12.0 months<br>-Median PFS: 2.7 months

							dermatofibrosarcoma protuberans, 1 osteosarcoma, 1 rhabdomyosarcoma, 1 synovial sarcoma)	-Adverse events: 58% of patients (any grade), 5% of patients experienced grade 3 or 4 adverse event
34	Monga V et al. (35)	2020	Retrospective study	-Pembrolizumab (47 patients)<br>-Nivolumab (6 patients)<br>-Ipilimumab (1 patient)<br>-Ipilimumab &nivolumab (27 patients)<br>-Other (7 patients)	-Pembrolizumab: 200 mg IV every 3 weeks<br>-Nivolumab 3 mg/kg IV every 2 weeks<br>-Combination: nivolumab 3 mg/kg & ipilimumab 1 mg/kg every 3 weeks (4 doses) followed by nivolumab (3 mg/kg) every 2 weeks (up to 2 years).	88	Metastatic STS	-Median PFS: 4.1 months<br>-Median OS: 19.1 months<br>-ORR: 23.9% (21 of 88)<br>-CR: 1 patient (undifferentiated pleomorphic sarcomas, on pembrolizumab)<br>-PR: 20 patients (7 undifferentiated pleomorphic sarcomas, 9 leiomyosarcomas, 1 alveolar soft part sarcomas, 1 fibroblastic sarcoma, 1 sclerosing epithelioid fibrosarcoma, 1 myxofibrosarcoma)<br>-SD: 28 patients
35	Ruohoniemi DM et al. (36)	2020	Retrospective study	Immune checkpoint inhibitors and radioembolization	-Patient 1: 1st immunotherapy – ipilimumab (1mg/kg) + nivolumab (240mg), 2nd immunotherapy – nivolumab (240mg)<br>-Patient 2: nivolumab 3mg/kg<br>-Patient 3: nivolumab 3mg/kg	3	STS with unresectable hepatic metastases	Best imaging response:<br>-Patient 1:<br>(a)Target tumour: PR<br>(b)Overall: immune-based stable disease<br>-Patient 2:<br>(a)Target tumour: SD<br>(b)Overall: immune-based stable disease<br>-Patient 3:<br>(a)Target tumour: PR<br>(b)Overall: immune-based stable disease
36	Yang J et al. (37)	2020	Phase 1	Toripalimab	(a) 3 mg/kg: 10 patients<br>(b) 10 mg/kg: 2 patients<br><br>-IV infusion once every 2 weeks	12	Advanced or recurrent alveolar soft part sarcoma	-No dose-limiting toxicity<br>-Median OS: 34.7 months<br>-Median PFS: 11.1 months<br>-Clinical response:<br>(a)Patients received 3 mg/kg: 1 complete response, 2 partial responses, 6 stable diseases

								(b)Patients received 10 mg/kg: 2 stable diseases<br>-ORR: 25%<br>-DCR: 91.7%
37	Quiroga D et al. (38)	2020	Retrospective study	-Nivolumab or<br>-Nivolumab and Ipilimumab or<br>-Pembrolizumab	-Nivolumab: 30 patients<br>-Nivolumab and Ipilimumab: 6 patients<br>-Pembrolizumab: 20 patients<br>-Median of 3 cycles	56	Advanced sarcomas: liposarcomas (n = 11), leiomyosarcomas (n = 7), synovial sarcoma (n = 4), chordoma (n = 4), spindle cell sarcoma (n = 4), osteosarcomas (n = 3), undifferentiated pleomorphic sarcoma (n = 3), other (n = 20)	-26 evaluable patients<br>-PR: 3/26 (1 liposarcomas treated with nivolumab, 1 inflammatory myofibroblastic sarcoma treated with nivolumab, 1 sarcomatoid carcinoma treated pembrolizumab)<br>-No complete responses<br>-Overall response rate 11.5%<br>-90-day PFS: 48.8%<br>-Median PFS: 11.3 weeks<br>-Median OS: 37.4 weeks
38	MarjaŃska A et al. (39)	2020	Clinical study	Nivolumab	Nivolumab 3mg/kg/2 weeks, 2 cycles	1	Localized extrasceletal chondrosarcoma myxoidale	Progression and death
39	Le Cesne A et al. (40)	2019	Phase 2	Pembrolizumab & Metronomic cyclophosphamide	-pembrolizumab: 200mg IV every 3 weeks (day 8 of a 21-day cycle)<br>-cyclophosphamide: orally 50 mg b.i.d. 1 week on / 1 week off	17	Metastatic and/or unresectable osteosarcomas	-Most frequent adverse events: nausea (grade I or II), anaemia, anorexia and fatigue<br>-Grade III or IV toxicities: fatigue, anaemia, lymphopenia and acute renal failure ((in 6 patients)<br>-15 patients assessable for efficacy<br>-tumour shrinkage: 4 patients (25%)<br>-Best clinical responses:<br>(a)PR: 1 patient (6.7%)<br>(b)SD: 5 patients (33.3%)<br>(c)PD: 8 patients (53.3%)<br>-6-month non-progression rate: 13.3%<br>-Median PFS: 1.4 months (95% CI = 1.0 months – 1.4 months)<br>-Median OS: 5.6 months (95% CI = 2.1 months – 12.1 months)
40	Wilky BA et al. (41)	2019	Phase 2	Axitinib & Pembrolizumab	Axitinib 5mg bd orally daily & pembrolizumab 200mg IV on day 8 and every 3 weeks for cycles of	33	Advanced sarcomas: 36% alveolar soft part sarcomas (n=12), 15% high-grade pleomorphic	- 3-month PFS (all evaluable patients): 65.6%<br>- 3-month PFS for alveolar soft part sarcomas: 72.7%

					6 weeks for up to 2 years for the first 5 patients. Then, patients treated with escalating doses of axitinib (2-10mg) & flat dose of pembrolizumab.		sarcoma (n=5), 12% uterine leiomyosarcoma (n=4), 6% non-uterine leiomyosarcoma (n=2), 6% dedifferentiated liposarcomas(n=2), 24% other (n=8)	- Most common grade 3 or 4 adverse events: hypertension (15%), autoimmune toxicities (15%), nausea or vomiting (6%), seizures (6%)<br>- Serious adverse events: 7 patients (21%) including autoimmune colitis, transaminitis, pneumothorax, haemoptysis, seizures, hypertriglyceridemia
41	Kelly CM et al. (42)	2019	Phase 2	Pembrolizumab & Epacadostat	-Epacadostat: 100mg bd<br>-Pembrolizumab: IV 200mg/dose every 3 weeks	29	Advanced sarcomas: leiomyosarcoma (17%), undifferentiated pleomorphic sarcoma (17%), myxofibrosarcoma (7%), liposarcoma (10.5%), epithelioid hemangioendothelioma (10.5%), angiosarcoma (3%), other sarcoma subtypes (35%)	-PR: 1 patient (leiomyosarcoma)<br>-SD: 13 patients<br>-PD: 15 patients<br>-Median PFS: 8 weeks<br>-Median OS: not estimable
42	D'Angelo SP et al. (43)	2018	Phase 2	Nivolumab ± Ipilimumab	-Nivolumab: 3 mg/kg every 2 weeks OR<br>-Nivolumab 3mg/kg & ipilimumab 1mg/kg every 3 weeks x 4 doses followed by nivolumab (3mg/kg) every 2 weeks.	85	Metastatic sarcoma	-76 evaluable patients for efficacy (38 patients received nivolumab monotherapy & 38 received nivolumab combined with ipilimumab)<br>-ORR in nivolumab monotherapy group: 5%<br>-ORR in combination therapy group: 16% (responses observed in undifferentiated pleomorphic sarcoma, leiomyosarcoma, myxofibrosarcoma, and angiosarcoma)
43	Gordon EM et al. (44)	2018	Retrospective study	Trabectedin & Nivolumab	-Trabectedin: 1.5 mg/m^2 (24hour infusion every 3 weeks)<br>-Nivolumab: 3mg/kg every 2 weeks	20	Advanced STS (7 undifferentiated pleomorphic sarcomas, 5 leiomyosarcomas, 2 synovial sarcomas, 4 myxoid liposarcoma, 2 chondrosarcoma)	Efficacy (17 patients):<br>-PR: 4 patients (1 undifferentiated pleomorphic sarcoma, 1 myxoid liposarcoma, 1 chondrosarcoma, 1 leiomyosarcoma)<br>-SD: 7 patients<br>-PD: 6 patients<br>-ORR: 23.5%

								-Median PFS: >11.6 months<br>-Median OS: >14.2 months
44	Toulmonde M et al. (45)	2017	Phase 2	Pembrolizumab & Metronomic cyclophosphamide	-Pembrolizumab: IV 200 mg every 3 weeks (day 8 of a 21-day cycle)<br><br>-Cyclophosphamide: orally 50 mg bd 1 week on / 1 week off	57	Advanced soft-tissue sarcoma (15 leiomyosarcomas, 16 undifferentiated pleomorphic sarcomas, 10 GIST and 16 other sarcomas)	-Most frequent adverse effects: fatigue (grade I or II), diarrhoea, anaemia.<br>- Grade III or IV toxic effects included: fatigue, oral mucositis, anaemia (in 10 patients).<br>-Clinical response assessment in 50 out of 57 patients<br>-6-month non-progression rates:<br>(a) Leiomyosarcoma: 0%<br>(b)Undifferentiated pleomorphic sarcoma: 0%<br>(c) Other sarcomas: 14.3%<br>(d) GIST: 11.1%<br>-Median PFS:<br>(a)Leiomyosarcoma: 1.4 months (95% CI, 1.2-1.4)<br>(b)Undifferentiated pleomorphic sarcomas: 1.4 months (95% CI, 1.1-4.0)<br>(c)Other sarcomas: 1.4 months (95% CI, 0.9-4.0)<br>(d) GIST: 1.4 months (95% CI, 0.9-5.3)<br>-Median OS:<br>(a) Leiomyosarcoma: 9.2months (95% CI, 2.4-15.9)<br>(b)Undifferentiated pleomorphic sarcomas: 5.6months (95% CI, 3.2-16.1)<br>(c) Other sarcomas: 7.1 months (95% CI, 2.0-16.3)<br>(d) GIST: not reached at the time of analysis
45	Weiss GJ et al. (46)	2017	Phase 1b	Pembrolizumab in combination with:<br>-Arm 1: Gemcitabine	Pembrolizumab: 2 mg/kg IV over 30 min every 21 days (prior to chemotherapy)	7	Metastatic sarcomas<br>-1 uterine leiomyosarcoma (Arm 4)<br>-1 fibromyxoid sarcoma (Arm 4)	OS (months):<br>-1 uterine leiomyosarcoma: 10.3<br>-1 fibromyxoid sarcoma: 19.2<br>-2 synovial sarcomas: 17.8, 13.3 respectively<br>-1 liposarcoma: 23.1

				-Arm 2: Gemcitabine & docetaxel<br>-Arm 3: Gemcitabine & nab-paclitaxel<br>-Arm 4: Gemcitabine & vinorelbine<br>-Arm 5: Irinotecan<br>-Arm 6: Liposomal doxorubicin	Arm 4: Gemcitabine 1000 mg/m^{2}and vinorelbine 25 mg/m^{2}on day 1 and day 8 every 21 days<br><br>Arm 6: Liposomal doxorubicin 30 mg/m^2 on day 1 every 21 days (total cumulative dose: 450 mg/m^{2}or 15 cycles if there are no dose reductions)		-2 synovial sarcomas (Arm 4)<br>-1 liposarcoma (Arm 6)<br>-1 malignant fibrious histiocytoma (Arm 6)<br>-1 clear cell sarcoma (Arm 6)	-1 malignant fibrious histiocytoma: 13.1<br>-1 clear cell sarcoma: 6.9<br><br>Best response:<br>-1 uterine leiomyosarcoma: NE<br>-1 fibromyxoid sarcoma: PD<br>-2 synovial sarcomas: PD<br>-1 liposarcoma: SD<br>-1 malignant fibrious histiocytoma: PD<br>-1 clear cell sarcoma: PD
46	Ben-Ami E et al. (47)	2017	Phase 2	Nivolumab	Nivolumab: 3 mg/kg IV on day 1 of each 2-week cycle	12	Advanced uterine leiomyosarcoma	-None responded to treatment.<br>-Overall median PFS:1.8 months
47	Tawbi HA et al. (48)	2017	Phase 2	Pembrolizumab	Pembrolizumab: 200 mg IV every 3 weeks	86	advanced soft tissue (n=40) and bone (n=40) sarcomas:<br>- STS: 10 undifferentiated pleomorphic sarcomas, 10 dedifferentiated liposarcomas, 10 synovial sarcoma, 10 leiomyosarcoma<br>- bone sarcomas: osteosarcoma, Ewing sarcoma, dedifferentiated chondrosarcoma	-86 were enrolled, 80 were evaluable for response<br>-Median follow-up for STS: 19.1 months<br>-ORR for STS: 18% [undifferentiated pleomorphic sarcoma ORR:40% (1 CR+3PR/10), 2 PR/10 in liposarcomas, 1PR/10 in synovial sarcoma, 0/10 in leiomyosarcoma]<br>-Median follow-up for bone sarcoma: 17.8 months<br>-ORR for bone sarcoma: 5% (1PR/22 in osteosarcoma, 1PR/5 in chondrosarcoma, 0/13 in Ewing sarcoma).
48	D'Angelo SP et al. (49)	2017	Phase 1b	Ipilimumab & Dasatinib	-Ipilimumab – dose escalation cohorts: 10 or 3 mg/kg every 3 weeks, followed by maintenance every 12 weeks<br>-Escalating doses of dasatinib: 70 mg daily, 100 mg daily, or 70 mg twice daily	28	GIST (n=20), other sarcomas (n=8)	- Doses: Dasatinib 70 mg/day with ipilimumab 10 mg/kg or dasatinib 140 mg/day with ipilimumab 3 mg/kg<br>- Response as per RECIST criteria: no PR or CR.<br>-Response as per Choi criteria: 7 of 13 evaluable patients with GIST had PR.<br>-Limited clinical efficacy.

49	Paoluzzi L et al. (50)	2016	Retrospective study	Nivolumab ± pazopanib (18 patients)	-IV nivolumab 3 mg/ kg every 2 weeks<br>-median number of cycles: 8<br>-pazopanib: 400–800 mg daily	28	Metastatic or locally advanced STS (n=24) or bone sarcomas (n=4)	-Most common side effect: grade 1–2 LFT elevations; 8/10 patients on pazopanib<br>-Grade 3–4 toxicity: 5 patients (colitis, LFT elevations, pneumonitis)<br>-24 patients evaluable for response<br>-PR: 3 patients (1 dedifferentiated chondrosarcoma, 1 epithelioid sarcoma (on pazopanib) & 1 maxillary osteosarcoma (on pazopanib)<br>-SD: 9 patients; 5/9 patients on pazopanib<br>-PD: 12 patients<br>-Clinical benefit: 50% of the evaluable patients
50	Maki RG et al. (51)	2013	Pilot trial	Ipilimumab	3 mg/kg IV every 3 weeks for 3 cycles	6	Advanced or metastatic Synovial sarcoma	PD: all patients (response rate 0%)

OS, overall survival; PFS, progression free survival; ORR, objective response rate; NE, not evaluable; PD, disease progression; SD, stable disease; PR, partial response, CR, complete response; DCR, disease control rate; ICI, immune checkpoint inhibitors; GIST, gastrointestinal stromal tumour; RT, radiation therapy; IP, intraperitoneal; RIT, radioimmunotherapy; IV intravenous; STS, soft tissue sarcoma; mAb, monoclonal antibody; AIM, anthracycline/ ifosfamide/ mesna; AD, anthracycline/ dacarbazine; NOS, not otherwise specified; TKIs, tyrosine-kinase inhibitors; nab-paclitaxel, nanoparticle albumin-bound paclitaxel

Table S2: Clinical experience of adoptive cellular therapy in sarcoma patients

No	Authors	Year	Type of study	Drug/Agent used	Treatment course	Number of patients	Type of sarcoma	Outcome
1	D'Angelo SP et al. (52)	2022	Pilot study	Letetresgene autoleucel (autologous T-cell therapy targeting NY-ESO-1 tumors)	-median transduced T cell dose: 4.6 x 10^9<br>-Cohort 1: 30 mg/m^2 fludarabine x 3days + 600mg/m^2 cyclophosphamide x 3days<br>-Cohort 2: 30 mg/m^2 fludarabine x 4days + 900mg/m^2 cyclophosphamide x 3days	23	Advanced or metastatic NY-ESO-1 myxoid/round cell liposarcoma	-20 patients were dosed with T cells (10 in each cohort)<br>-Investigator-assessed ORR:<br>(a)Cohort 1: 20%<br>(b)Cohort 2: 40%<br>-mPFS:<br>(a)Cohort 1: 5.4 months<br>(b)Cohort 2: 8.7 months
2	Kohli K et al. (53)	2021	Phase 1	NY-ESO-1–specific endogenous T cells (ETC) following cyclophosphamide conditioning	Cell dose: 19 – 27.5 x 10^9 (target cell doses of 10^{10}/m^2)	4	2 synovial sarcomas, 2 myxoid/ round cell liposarcoma	-Well tolerated.<br>-Patient 1 - myxoid/ round cell liposarcoma: SD, resected. Progression 1 year post ECT.<br>-Patient 2 - synovial sarcomas: 20% reduction in a liver lesion at 4 weeks. Progression at 8-10 weeks.<br>-Patient 3 - synovial sarcomas: 36% reduction in a lung lesion at 4 weeks. Progression at 8-10 weeks.<br>-Patient 4 - myxoid/ round cell liposarcoma: 16% reduction in a mediastinal lesion. Progression at 8-10 weeks.
3	Shi J et al. (54)	2020	Retrospective study	Adjuvant chemotherapy ± TILs therapy	-Group 1: received adjuvant chemotherapy – MAP regimen (40 patients)<br>-Group2: received adjuvant chemotherapy & TILs therapy (40 patients)	80	Osteosarcoma	-Median DFS: 55.5 months in group 1 vs 65.3 months in group 2<br>-Median OS: 80.4 months in group 1 vs 95.8 months in group 2

4	Zhou X et al. (55)	2020	Retrospective study	TIL therapy & anti-PD1 therapy	-Anti-PD1 therapy infusions: nivolumab 3 mg/kg/cycle<br>-TILs were transfused in the first cycles of nivolumab<br>-Average number of TILs: $5x10^9$ cells (range $3\text{-}8x10^9$)	60	Chemotherapy-resistant metastatic osteosarcoma	-Most common adverse effects: fever, fatigue, rash, anorexia, leukopenia, anaemia<br>-ORR: 36.67% (22/60)<br>-CR: 2 patients<br>-PR: 20 patients<br>-Median PFS: 5.75 months<br>-Median OS: 13.6 months
5	Wang C et al. (56)	2020	Retrospective study	Anti-PD1 therapy ± TILs therapy	-Group 1: anti-PD1 therapy (30 patients)<br>-Group 2: anti-PD1 + TILs therapy (30 patients)<br>-Both groups: nivolumab 3mg/kg/cycle (max dose 240mg/cycle)	60	Metastatic osteosarcoma	-ORR: 6.67% Group1 vs 33.3% Group2<br>-Median PFS: 3.8 months Group1 vs 5.4 months Group2<br>-Median OS: 6.6 months Group1 vs 15.2 months Group2
6	Nowicki TS et al. (57)	2019	Pilot trial	Transgenic ACT with DC vaccination ± ipilimumab	-ESO cohort: Adoptive transfer of autologous NY-ESO-1-specific TCR transgenic lymphocytes & NY-ESO-1 peptide-pulsed DC vaccination in HLA-A2.1-positive subjects<br>-INY cohort: ACT & DC vaccination & ipilimumab	8	-HLA-A*0201–positive patients with NY-ESO-1–positive tumours<br>-5 synovial sarcomas, 1 osteosarcoma, 1 liposarcoma, 1 malignant peripheral nerve sheath tumour, (2 patients with metastatic melanoma were enrolled as well)	-Evidence of tumour regression:<br>(a)ESO cohort: 4/6 patients (66%)<br>(b)INY cohort: 2/4 patients (50%)<br>-No clinical benefit of additional ipilimumab.
7	Ramachandran I et al. (58)	2019	Phase I/II therapy	NY-ESO-1 SPEAR T-cells	-Median transduced cell dose overall: 2.67×10^9<br>-Cohort 1: fludarabine 30 mg/ m^2/day × 4 days & cyclophosphamide 1800 mg/ m^2 /day × 2 days<br>-Cohort 2: fludarabine 30 mg/ m^2/day × 4 days	42	Synovial sarcoma patients, HLA-A*02 positive<br>(a)tumours expressed NY-ESO-1 antigen<br>(b)Antigen expression by IHC:<br>-Cohort 1/ 3/ 4: 2+ or 3+ in ≥50% of tumour cells	-CR: 1 patient<br>-PR: 14 patients<br>-SD: 24 patients<br>-PD: 3 patients

					& cyclophosphamide 1800 mg/ m^2 /day × 2 days<br>-Cohort 3: cyclophosphamide 1800 mg/ m^2 /day × 2 days<br>-Cohort 4: fludarabine 30 mg/ m^2/day × 3 days & cyclophosphamide 600 mg/ m^2/day × 3 days		-Cohort 2: 1+ in ≥ 1%, but not ≥50% of tumour cells expressing 2+ or 3+	
8	B.A. Van Tine et al. (59)	2019	Phase 1	ADP-A2M4, SPEAR T-cells against MAGE-A4 peptide	Median T-cell dose: $9.7x10^9$ ($4.49-9.98x10^9$)	10	MAGE-A4$^+$ inoperable or metastatic synovial sarcoma, HLA-A*02 patients	-No dose limiting toxicity<br>-PRs: 3 patients and 1 patient with unconfirmed PR at week 6<br>-SD: 3 patients<br>-PD: 1 patient<br>-2 patients were not evaluated
9	Lu YC et al. (60)	2017	Phase 1/2	Autologous CD4+ T-cells - transduced with MAGE-A3 TCR	-Synovial sarcoma patient: $30x10^9$ cells twice<br>-Osteosarcoma patient: $123x10^9$ cells + IL2 (720,000 IU/kg)	2	-Metastatic synovial sarcoma<br>-Metastatic osteosarcoma	-No treatment-related deaths<br>-Response:<br>(a)Synovial sarcoma: no response<br>(b)Osteosarcoma: partial response (4 months duration)
10	Thiel U et al. (61)	2017	Clinical study	HLA-A*02:01/peptide-specific allorepertoire-derived CD8$^+$ T cells	-Patient 1: $4.8x10^5$/kg HLA-A*02:01$^-$ allorestricted donor-derived wild type CD8$^+$ T cells<br>-Patient 2: $8.2x10^6$/kg HLA-A*02:01$^-$ donor derived<br>-Patient 3: $6x10^6$/kg autologous allorestricted TCR transgenic CD8$^+$ T cells	3	Refractory HLA-A2$^+$ Ewing sarcoma patients	-Patients 1 & 3: slow progression<br>-Patient 2: partial metastatic regression
11	Ahmed N et al. (62)	2015	Phase 1/2	HER2-specific CAR T cell	escalating doses of HER2-CAR T cells from $1x10^4/m^2$ to $1x10^8/m^2$	19	Refractory or recurrent metastatic HER2 positive sarcomas (16 osteosarcoma, 1 Ewing	-No dose-limiting toxicity<br>-Clinical response assessment in 17 out of 19 patients:

					1 infusion: 13 patients<br>2 infusions: 4 patients<br>4 infusions: 1 patient<br>9 infusions: 1 patient		sarcoma, 1 primitive neuroectodermal tumor, 1 desmoplastic small round cell tumor)	Stable disease for 12 weeks to 14 months: 4 patients (excision of the tumor in 3 patients, 90% necrosis in one of them)<br>-Median overall survival: 10.3 months (range, 5.1 - 29.1 months)
12	Robbins PF et al. (63)	2015	Pilot trial	Autologous TCR-transduced T cells (against NY-ESO-1)	5.5×10^{10} T cells (range $0.9–13\times10^{10}$) + 720,000 iU/kg of interleukin-2	18	Metastatic synovial cell sarcoma (HLA-*0201 positive, NY-ESO-1–positive)	-No toxicities from T cells (transient neutropenia and thrombocytopenia due to preparative chemotherapy regimen and the transient toxicities induced by IL-2).<br>-ORR: 11 patients (61%)<br>-Partial responses lasted: 3 to 18 months<br>-Overall 3-year survival rate: 38%<br>-Overall 5-year survival rate: 14%
13	Tonn T et al. (64)	2013	Phase 1	NK-92 cells	-NK-92 starting cell dose (10^9 / m^2):<br>(a)STS - Patient 1: 0.85<br>(b)STS - Patient 2: 3<br>(c)Rhadomyosarcoma: 1<br>(d)OS: 1<br><br>-NK-92 total cell dose (10^9):<br>(a)STS - Patient 1: 2.4<br>(b)STS – Patient 2: 9.4<br>(c)Rhadomyosarcoma: 4<br>(d)Osteosarcoma: 2.6	4	Advanced, treatment-resistant malignancies:<br>2 STS<br>1 rhadomyosarcoma<br>1 osteosarcoma	-No infusion-related or long-term side effects<br>-Response to NK cell therapy:<br>(a)STS - Patient 1: PD<br>(b)STS - Patient 2: PD<br>(c)Rhadomyosarcoma: PD<br>(d)Osteosarcoma: PD<br>-OS (in days):<br>(a)STS - Patient 1: 262<br>(b)STS - Patient 2: 13<br>(c)Rhadomyosarcoma: 42<br>(d)Osteosarcoma: 99
14	Montagna D et al. (65)	2012	Pilot study	Autologous ex vivo-generated anti-tumour-specific CTL	-Patient with kidney/undifferentiated STS: 5 infusions / 0.15-0.35×10^9 CTL per each infusion<br>-Uterus/stromal sarcoma patient: 17 infusions / 0.2-4.0×10^9 CTL per each infusion	2	Soft tissue sarcoma (1 kidney/undifferentiated STS, 1 Uterus/stromal sarcoma)	-No acute or late adverse events.<br>-No grade III/IV events related to CTL infusion.

					-Low-dose IL-2 (1 M U/day) s.c. for 2 weeks following each CTL infusion			
15	Dillman RO et al. (66)	2003	Phase II	Autologous Activated Lymphocytes+ Cimetidine	-Number of cells infused: $0.82x10^9$ - $12.8x10^9$<br>-Cimetidine dose: 600 mg po qid	2	Sarcoma	Objective tumour response: 0/2
16	Mazumder A et al. (67)	1984	Phase I	PHA activated autologous PBL	-Number of infusions: 7-9<br>-A total number of cells infused: 1.6 – 7.2 x 10^{10}	6	Metastatic sarcoma (2 synovial sarcomas, 1 epithelioid sarcoma, 1 osteosarcoma, 1 fibrosarcoma, 1 undifferentiated sarcoma)	-Toxicity included: fever & chills, headaches, nausea & vomiting, erythrocyte transfusion<br>-Evidence of migration of activated cells to tumour, lungs, liver and spleen

OS, overall survival; PFS, progression free survival; ORR, objective response rate; STS, soft tissue sarcomas; CTL, cytotoxic T lymphocytes; s.c., subcutaneously; CR, complete response; PR: partial responses; SD, stable disease; PD, progressive disease; TILs, tumour-infiltrating lymphocytes; DFS, disease-free survival; NK, natural killer; TCR, T-cell receptor; CAR, Chimeric Antigen Receptor; PHA, phytohemagglutinin; PBL, peripheral blood lymphocytes

Table S3: Clinical experience of cancer vaccines in sarcoma patients

No	Authors	Year	Type of study	Drug/Agent used	Treatment course	Number of sarcoma patients	Type of sarcoma	Outcome
1	DeMaria PJ et al. (68)	2021	Phase 2	Yeast-Brachyury Vaccine (GI-6301) & RT vs placebo & RT	-3 doses of GI-6301 or placebo followed by RT, then vaccine or placebo continuation until progression<br>-GI-6301: 80 × 10^7 yeast cells	24	Locally advanced unresectable chordoma (11 patients in the vaccine arm and 13 patients in the placebo arm)	-PR: 1 in each arm<br>-No CR<br>-Median PFS: 20.6 months vaccine arm vs 25.9 months placebo.<br>-No difference in overall response rate, early discontinuation.
2	Dhir Aditi et al. (69)	2021	Phase 1	Autologous DC vaccination	-5+3 dose-escalation schema<br>-Three dose levels: 3, 6, and 12 x 10^6 DCs per treatment<br>-Intradermal administration in imiquimod-treated skin	19	Recurrent/refractory sarcomas (children & adults, range 13-75 years)	-No treatment related dose limiting toxicity<br>-Median PFS: 9.5 months
3	Somaiah N et al. (70)	2020	Phase 1b	CMB305 (Lentiviral-Based) prime -boost vaccine	3 + 3 dose-escalation design followed by an expansion with CMB305 alone or combined with oral metronomic cyclophosphamide or intratumoral injections glucopyranosyl lipid A	64	-NY-ESO-1 expressing tumours<br>-Locally Advanced, Relapsed, or Metastatic Sarcomas (myxoid/ round cell liposarcoma, synovial sarcomas, other)	-Most common adverse events: fatigue, nausea, injection-site pain<br>-DCR: 61.9%<br>-OS: 26.2 months
4	Kelly CM et al. (71)	2020	Phase 2	T-VEC & Pembrolizumab	-pembrolizumab: IV 200mg flat dose<br>-T-VEC (first dose, ≤4 mL × 10^6 PFU/mL; second and subsequent doses, ≤4 mL × 10^8 PFU/mL) injected into palpable tumor site(s)<br>-Both drugs administered on day 1 of each 21-day cycle	20	Locally advanced or metastatic sarcoma:<br>-5 leiomyosarcomas<br>-3 angiosarcomas<br>-2 undifferentiated pleomorphic sarcomas<br>-3 undifferentiated or unclassified sarcoma<br>-7 other histologic subtypes (including chondrosarcoma, extraskeletal myxoid	-Incidence of grade 3 treatment-related adverse events: 20% (4 patients)<br>-Best ORR (at 24 weeks): 30% (1 patient with delayed response at 32 weeks increased the ORR to 35%)<br>- Objective response:<br>(a)CR: 0<br>(b)PR: 7 patients (35%)<br>(c)SD: 7 patients (35%)

							chondrosarcoma, malignant peripheral nerve sheath tumour, epithelioid sarcoma, alveolar soft part sarcoma, myxofibrosarcoma, and synovial sarcoma)	(d)PD: 6 (30%)<br>-Median time to response: 14.4 weeks (range, 6.6-31.9 weeks)<br>-Median duration of response: 56.1 weeks (range, 49.4-87.0 weeks)<br>-5 histologic subtypes with PR: cutaneous angiosarcoma of head and neck (n = 2), undifferentiated pleomorphic sarcomas (n = 2), myxofibrosarcoma (n = 1), epithelioid sarcoma (n = 1), sarcoma unclassified (n = 1)
5	Fedorova L et al. (72)	2019	Phase 1/2	Monocyte-derived DC-based vaccines	Dose: 2 × 10^6 DCs in 100 µl of cryopreservation medium. Intradermal administration (arm, near the axillary lymph node) every 3 ± 1 weeks.	25	High-risk sarcoma<br>(25 sarcoma patients enrolled in the trial but DC vaccine was administered in 11 patients and 9 received at least 6 doses).	Outcome of the 9 patients who received at least 6 doses:<br>-Patient 1 - Ewing: metastatic progression<br>-Patient 2 - Osteosarcoma: metastatic progression<br>-Patient 3 - synovial sarcomas: metastatic progression<br>-Patient 4 - Ewing: Locoregional progression<br>-Patient 5 - Alveolar rhabdomyosarcoma: CR<br>-Patient 6 - Osteosarcoma: progression<br>-Patient 7 - Embryonal rhabdomyosarcoma: PR<br>-Patient 8 - Osteosarcoma: PD<br>-Patient 9 - osteoblastic osteosarcoma: SD
6	Miwa S et al. (73)	2017	Phase 1/2	Autologous tumor lysate pulsed DCs	-5x10^6 cells/1 mL of normal saline via s.c. injections into the inguinal or axillary region.<br><br>-6 weekly injections	37	Metastatic or recurrent bone and soft tissue sarcomas<br>-17 patients with bone tumors (14 osteosarcoma, 3 chondrosarcomas)<br>-20 patients with soft tissue tumors (5 malignant fibrous	-No severe adverse events or deaths<br>-Clinical response assessment in 35 out of 37 patients - 8 weeks after the DC injection:<br>(a)Tumor progression: 28 patients (80%)

							histiocytomas, 4 clear cell sarcomas, 2 synovial sarcomas, 3 leiomyosarcoma, 1 Ewing sarcoma, 1 liposarcoma, 1 alveolar soft part sarcoma, 1 angiosarcoma, 1 ependymoma, 1 malignant peripheral nerve sheath tumour)	(b)Stable disease: 6 patients (17.1%)<br>(c)Partial response: 1 patient (2.9%)<br>-3-year overall survival rate: 42.3%<br>-3-year progression-free survival rate: 2.9%
7	Merchant MS et al. (74)	2016	Phase 1/2	Adjuvant immunotherapy: autologous lymphocytes, tumor lysate/KLH –pulsed dendritic cell vaccinations ± recombinant human IL7	Cohort 1 (n=5):<br>-autologous lymphocyte infusion, day 2<br>-DC vaccines D2,16,30,44,58,72<br><br>Cohort 2 (n=24):<br>-autologous lymphocyte infusion, day 2<br>-DC vaccines D2,16,30,44,58,72<br>CYT107: 20mcg/kg SQ D0,14,28,42<br><br>6 DC vaccines were injected: 3 s.c. sites (1×10^7 cells/site), and 3 ID sites (1×10^6 cells/site)	-43 enrolled<br>-29 received immunotherapy	newly diagnosed metastatic or recurrent pediatric sarcoma: Ewing sarcoma, rhabdomyosarcoma, desmoplastic small round cell tumour, synovial sarcoma, and undifferentiated sarcoma	-Intent-to-treat analysis of all patients enrolled demonstrates: 5-year OS of 51% and PFS of 32%.<br>-Outcomes varied:<br>(a)Ewing sarcoma/Rhabdomyosarcoma: 5-year OS 63%, PFS 40%<br>(b) Other sarcomas: 5-year OS 0%, PFS 0%
8	Ghisoli M et al. (75)	2016	Prospective study	Vigil vaccine	Once monthly by intradermal injection ($1\times10e^6$ cells/injection to $1\times10e^7$ cells/injection)	30	Metastatic Ewing's Sarcoma	-16 Vigil-treated patients compared to 14 non-Vigil-treated patients.<br>-73% 1-year survival for Vigil-treated patients vs 23% in not treated with Vigil patients.<br>-17.2-month difference in overall survival between the Vigil and no Vigil patient groups.

9	Krishnadas DK et al. (76)	2015	Phase 1	Decitabine (DAC) /dendritic cell vaccine targeting MAGE-A1, MAGE-A3 and NY-ESO-1	Week 1: DAC 10 mg/m^2/day for 5 days and weeks 2 and 3: DC vaccine once weekly (3-10 × 10^6 cells based on patient weight)	5	Children with relapsed or therapy – refractory sarcoma (2 Ewing sarcoma, 2 osteosarcomas, 1 rhabdomyosarcoma)	2 patients were evaluable (2 osteosarcoma and 1 ES patients did not receive therapy due to further progression between the time of enrolment and the start of therapy):<br>-1 Ewing sarcoma: progression, grade 4 neutropenia at cycle 2<br>-1 Rhabdomyosarcoma: progression, grade 4 neutropenia at cycle 2
10	Cripe TP et al. (77)	2015	Phase 1	Intratumoral Pexa-Vec (JX-594)	Total Pexa-Vec dose (pfu) in volume:<br>-Pubis: 7.65×10^8 25 ml<br>-Lung: 7.53×10^8 25 ml	1	Ewing sarcoma	Response to Pexa-Vec at day 22:<br>-Pubis: stable disease<br>-Lung: progressive disease<br>-New: yes
11	Takahashi R et al. (78)	2013	Phase 2	Personalized peptide vaccination (31 peptides)<br><br>-in combination with ChT: 3 patients<br><br>- in combination with RT: 2 patients	s.c. administration of a maximum of 4 HLA-matched peptides (3 mg/each peptide) weekly for 6 weeks and bi-weekly thereafter	20	Refractory bone and soft tissue sarcoma (4 leiomyosarcoma, 3 osteosarcoma, 3 myxofibrosarcoma, 3 synovial sarcomas, 2 liposarcomas, 1 malignant neurinoma, 1 epithelioid sarcoma, 1 clear cell sarcoma, 1 chondrosarcoma, 1 alveolar soft part sarcoma)	-No severe adverse events related to personalized peptide vaccination<br>-Median overall survival time: 9.6 months<br>-Median progression free survival time: 4 months<br>-Best clinical responses:<br>(a)CR: none<br>(b)PR: none<br>(c)SD: 6 patients<br>(d)PD: 14 patients
12	Coosemans A et al. (79)	2013	Phase I/II	WT1 mRNA-electroporated DCs vaccine	Number of injected DCs:<br>(a)Patient 1: 21.5×10^6<br>(b)Patient 2: 8.78×10^6<br>(c)Patient 3: 5.9×10^6	3	Uterus leiomyosarcoma (patient 1&2: HLA-A2-positive; patient 3: HLA-A2-negative)	-PFS with immunotherapy:<br>(a)Patient 1: 3 months<br>(b)Patient 2: 2 months<br>(c)Patient 3: 0 months<br>-Survival after end of immunotherapy:<br>(a)Patient 1: 4 months<br>(b)Patient 2: 4 months

								(c)Patient 3: 3 months<br>-OS:<br>(a)Patient 1: 43 months<br>(b)Patient 2: 22 months<br>(c)Patient 3: 20 months
13	Himoudi N et al. (80)	2011	Phase 1	Autologous DCs matured with autologous tumour lysate and KLH	-3 weekly vaccines, max of 6 vaccinations<br>-Administered ID near inguinal lymph nodes<br>-DC dose escalation:<br>(a) level 1: 1×10^5DC/kg<br>(b) level 2: 5×10^5/kg<br>(c) level 3: 1×10^6/kg<br>- Systemic IL-2: $3x10^6$ IU/ m^2/day s.c. (2 divided doses) after the 3rd DC course and subsequent courses (for 5 days).	14	-Relapsed osteosarcomas (13 patients recruited, 12 received vaccination)<br>-Ewing sarcoma	-Max achievable dose: 5×10^5/kg<br>-No significant toxicity.<br>-No evidence of clinical benefit.
14	Karbach J et al. (81)	2010	Phase 1	NY-ESO-1 peptide, CpG 7909 & Montanide ISA-51 vaccine	-Each day of vaccination: 100 µg of NY-ESO-1 peptide, 1 mg CpG 7909, 0.5 ml of Montanide ISA-51<br>-s.c. injections	1	Retroperitoneal liposarcoma	-Best response: no evidence of disease (of note, patient was enrolled in the study in a no evidence of disease status)<br>-Time to progression: 5,73 months
15	Suminoe A et al. (82)	2008	Clinical study	DCs pulsed with tumour-specific synthetic peptides or tumour lysates and KLH (post conventional ChT/RT & autologous PBSCT)	DC number/dose:<br>-Ewing sarcoma: $3x10^6$<br>-Synovial sarcoma: $3x10^6$<br>Number of administrations:<br>-Ewing sarcoma: 8<br>-Synovial sarcoma: 7	2	Refractory and/or relapsed sarcoma:<br>-Ewing sarcoma<br>-Synovial sarcoma	Ewing sarcoma: CR (lasting for 77 months)<br>Synovial sarcoma: SD (4 weeks) → PD
16	Dillman R et al. (83)	2004	Phase I/II	Irradiated Autologous Tumour-Cell Vaccines	Once a week for 3 weeks, then once a month for 5 months - s.c. injections	25	Sarcoma patients	-23 evaluable patients<br>-Well tolerated (no grade III or IV toxicities)<br>-Median failure-free survival: 3.8 months

								-Median OS: 11.2 months
17	Hernando JJ et al. (84)	2002	Phase I	Vaccines with DC pulsed with KLH and autologous tumour antigens	-Number of vaccinations:<br>(a)Patient 1: 3<br>(b)Patient 2: 6<br>-DC dose:<br>(a)Patient 1: 1.5-2×10^6<br>(b)Patient 2: 1.2×10^6	2	Uterine sarcoma	Progression free interval under vaccination:<br>(a)Patient 1: 3 months<br>(b)Patient 2: 6 months
18	Geiger JD et al. (85)	2001	Phase I	Vaccine with DCs pulsed with tumour cell lysates and KLH	DC dose:<br>1×10^6 – $1x10^7$	8	Fibrosarcoma, inflammatory myofibroblastic, osteosarcoma hepatic sarcoma, desmoplastic round cell, clear cell sarcoma, 2 Ewing sarcomas	No significant toxicities<br>Tumor response:<br>-Fibrosarcoma: PR<br>-Inflammatory myofibroblastic: SD<br>-Osteosarcoma: PD<br>-Hepatic sarcoma: PD<br>-Desmoplastic round cell: PD<br>-Clear cell sarcoma: PD<br>-Ewing sarcoma (1): PD<br>-Ewing sarcoma (2): PD
19	Campanacci M et al. (86)	1981	Retrospective study	Irradiated autologous tumour cells vaccine	-2-6 injections, intramuscular	16	Osteosarcoma	-2/16: disease free 90 months post-surgery<br>-1/16 local recurrence (no metastases 80 months later)<br>-13/16 metastases

OS, overall survival; PFS, progressive-free survival; DCR, disease control rate; PR, partial response; SD, stable disease; PD, progressive disease; CR, complete remission; DCs, dendritic cells; ChT, chemotherapy; RT, radiotherapy; T-VEC, Talimogene laherparepvec; KLH, keyhole limpet hemocyanin; s.c, subcutaneous; ID, intradermal; Pexa-Vec, pexastimogene devacirepvec; WT1, Wilms' Tumor Gene 1; CCID50, 50% cell-culture infectious dose; PBSCT, peripheral blood stem cell transplantation; VP, virus particles; MTX, methotrexate; HA, hemagglutinating activity; C. Parvum, Corynebacterium Parvum; LNs, lymph nodes

Table S4 Clinical experience of Interleukin 2 or Interferon-γ use in sarcoma patients

No	Authors	Year	Type of study	Drug/Agent used	Treatment course	Number of sarcoma patients	Type of sarcoma	Outcome
1	Zhang S et at. (87)	2019	Phase 0	IFNγ	Systemic s.c. administration: 2-4 weekly injections of IFNγ 100mcg/ m^2	8	-Synovial sarcoma<br>-Myxoid/round cell liposarcoma	TME changes (tumour-surface MHC-I expression and T-cell infiltration) due to IFNγ.
2	Meazza C et al. (88)	2017	Prospective study	IL-2, HDMTX, doxorubicin, cisplatin, ifosfamide, vincristine, surgery/RT ± LAK cells reinfusion	-r-IL-2:<br>9×10^6 IU/sqm/day	35	Primary metastatic osteosarcoma in children	-3-year event-free survival rate: 34.3%<br>-3-year overall survival rate: 45.0%<br>-24 patients had a progression or relapse - a median 10 months (range 4–50 months) after their diagnosis<br>-23 died a median 18 months (range 8–150 months) after their diagnosis
3	Miki Y et al. (89)	2013	Retrospective study	rIL-2 + RT ± ChT (Docetaxel)	-rIL-2 alone (4 patients), rIL-2 + docetaxel (2 patients)<br>-Total dose: $40x10^4$ – $2450x10^4$ units (daily dose of $35x10^4$ –$80x10^4$ units)	6	Angiosarcoma of the face and scalp	Median survival time: 18.65 months
4	Ogawa K et al. (90)	2012	Retrospective study	rIL2 ± ChT and/or RT and/or Surgery	-Total dose of rIL-2: $30x10^5$ - $194x10^5$ units<br>-systemic administration ($30x10^5$ - $119x10^5$ units) or intratumoral injection ($3.5x10^5$ to $194x10^5$ units)	35	Angiosarcoma of the scalp and face	Overall survival 2-year rate: 25.7%
5	Ohguri T et al. (91)	2005	Retrospective study	rIL-2 & curative RT & limited surgery (5 patients) & concomitant	Administration:<br>-transcatheter arterial administration (10 patients): daily dose $7x10^5$	20	Angiosarcoma of the scalp	-Median OS: 36.2 months<br>-Local recurrence-free: 11.1 months

				paclitaxel (2 patients)	– $8x10^5$ U (total dose of $192x10^5$ - $420x10^5$ U)<br>-systemic administration (11 patients): daily dose $3.5x10^5$ - $8x10^5$ U (total dose of $28x10^5$ -$416x10^5$ U)<br>-intratumoral injection (10 patients): daily dose $4x10^5$ - $8x10^5$ U (total dose $24x10^5$ - $640x10^5$ U)			-Distant metastasis-free survival: 17.8 months<br>-Prolongation of distant metastasis-free survival rates with combination of arterial or intratumoral administration with systematic administration.
6	Sasaki R et al. (92)	2002	Retrospective study	rIL-2 & RT	-Intratumoral injection (18 patients), transcatheter arterial administration (2 patients), systemic administration (2 patients)<br>-Total dose: $100x10^4$ – $1000x10^4$ units (7 patients), $1100x10^4$ - $2800x10^4$ units (13 patients)	20	Angiosarcomas	Median survival time:<br>-Patients received $100x10^4$ – $1000x10^4$ units: 5 months<br>-Patients received $1100x10^4$ - $2800x10^4$ units: 10 months<br>-Total amounts of rIL-2 had no prognostic significance
7	Le Cesne A et al. (93)	1999	Pilot study	Combination of r-IL-2 & DOX	1 injection of DOX (70 mg/m^2) and 3 weeks later a combination of r-IL-2 (18 MIU/m^2 days 1-5 s.c.) and DOX (same dose)<br>-Arm 1: DOX administered 3-4 h after the first r-IL-2 injection<br>-Arm 2: DOX administered 2 days after the last r-IL-2 injection	15	-12 soft tissue sarcomas<br>-2 osteosarcomas<br>-1 desmoplastic tumour	-No response after DOX alone<br>-After DOX & IL-2: objective response in 2 soft tissue sarcoma patients (one in each arm) - 8 and 5 months

r-IL-2, recombinant interleukin-2; DOX, doxorubicin; s.c., subcutaneous; HDMTX, high-dose methotrexate; LAK, lymphokine-activated killer; RT, radiotherapy; TME, tumour microenvironment; PR, partial response; SD, stable disease; PD, progressive disease; OS, overall survival

References

1. Italiano A, Bessede A, Pulido M, Bompas E, Piperno-Neumann S, Chevreau C, et al. Pembrolizumab in Soft-Tissue Sarcomas with Tertiary Lymphoid Structures: A Phase 2 Pembrosarc Trial Cohort. *Nat Med* (2022) 28(6):1199-206. Epub 20220526. doi: 10.1038/s41591-022-01821-3.
2. Gordon EM, Chawla SP, Chua-Alcala VS, Kim TT, Adnan N, Sekhon S, et al. Five-Year Results of a Phase 2 Trial Using Ipilimumab (I), Nivolumab (N), and Trabectedin (T) for Previously Untreated Advanced Soft Tissue Sarcoma (Nct03138161). *Journal of Clinical Oncology* (2022) 40(16_suppl):11573-. doi: 10.1200/JCO.2022.40.16_suppl.11573.
3. Wagner MJ, Zhang Y, Cranmer LD, Loggers ET, Black G, McDonnell S, et al. A Phase 1/2 Trial Combining Avelumab and Trabectedin for Advanced Liposarcoma and Leiomyosarcoma. *Clin Cancer Res* (2022) 28(11):2306-12. doi: 10.1158/1078-0432.Ccr-22-0240.
4. Toulmonde M, Brahmi M, Giraud A, Chakiba C, Bessede A, Kind M, et al. Trabectedin Plus Durvalumab in Patients with Advanced Pretreated Soft Tissue Sarcoma and Ovarian Carcinoma (Tramune): An Open-Label, Multicenter Phase Ib Study. *Clin Cancer Res* (2022) 28(9):1765-72. doi: 10.1158/1078-0432.Ccr-21-2258.
5. Toulmonde M BM, Giraud A, Bessede A, Kind M, Toulza E, et al. Lba67 Tramune, a Phase Ib Study Combining Trabectedin and Durvalumab, Results of the Expansion Cohort in Patients with Advanced Pretreated Soft Tissue Sarcomas. *Annals of Oncology* (2020) 31(S1199). doi: https://doi.org/10.1016/j.annonc.2020.08.2308.
6. Salkeni MA, Conley AP, Chen JL, Davis EJ, Burgess MA, Razak ARA, et al. A Phase 2 Study of Anti-Pd-L1 Antibody (Atezolizumab) in Grade 2 and 3 Chondrosarcoma. *Journal of Clinical Oncology* (2022) 40(16_suppl):11528-. doi: 10.1200/JCO.2022.40.16_suppl.11528.
7. D'Angelo SP, Richards AL, Conley AP, Woo HJ, Dickson MA, Gounder M, et al. Pilot Study of Bempegaldesleukin in Combination with Nivolumab in Patients with Metastatic Sarcoma. *Nat Commun* (2022) 13(1):3477. Epub 20220616. doi: 10.1038/s41467-022-30874-8.
8. Somaiah N, Conley AP, Parra ER, Lin H, Amini B, Solis Soto L, et al. Durvalumab Plus Tremelimumab in Advanced or Metastatic Soft Tissue and Bone Sarcomas: A Single-Centre Phase 2 Trial. *Lancet Oncol* (2022) 23(9):1156-66. Epub 20220804. doi: 10.1016/s1470-2045(22)00392-8.
9. Adnan N, Sekhon S, Chawla SP, Kim TT, Chua-Alcala VS, Fernando M, et al. Gallant: A Phase 2 Study Using Metronomic Gemcitabine, Doxorubicin, Nivolumab, and Docetaxel as Second/Third-Line Therapy for Advanced Sarcoma (Nct04535713). *Journal of Clinical Oncology* (2022) 40(16_suppl):11518-. doi: 10.1200/JCO.2022.40.16_suppl.11518.
10. Cousin S BC, Guegan J, Valentin T, Bahleda R, Metges J, et al., editor. 1494p - Regomune - a Phase II Study of Regorafenib + Avelumab in Solid Tumors: Results of the Soft Tissue Sarcoma (Sts) Cohort. *ESMO Congress 2022* 2022
11. Jones RL, Ravi V, Brohl AS, Chawla S, Ganjoo KN, Italiano A, et al. Efficacy and Safety of Trc105 Plus Pazopanib Vs Pazopanib Alone for Treatment of Patients with Advanced Angiosarcoma: A Randomized Clinical Trial. *JAMA Oncol* (2022) 8(5):740-7. doi: 10.1001/jamaoncol.2021.3547.
12. Tian Z, Dong S, Yang Y, Gao S, Yang Y, Yang J, et al. Nanoparticle Albumin-Bound Paclitaxel and Pd-1 Inhibitor (Sintilimab) Combination Therapy for Soft Tissue Sarcoma: A Retrospective Study. *BMC Cancer* (2022) 22(1):56. Epub 20220112. doi: 10.1186/s12885-022-09176-1.
13. Livingston MB, Jagosky MH, Robinson MM, Ahrens WA, Benbow JH, Farhangfar CJ, et al. Phase II Study of Pembrolizumab in Combination with Doxorubicin in Metastatic and Unresectable

Soft-Tissue Sarcoma. *Clin Cancer Res* (2021) 27(23):6424-31. Epub 20210902. doi: 10.1158/1078-0432.Ccr-21-2001.
14. Wagner MJ, Othus M, Patel SP, Ryan C, Sangal A, Powers B, et al. Multicenter Phase II Trial (Swog S1609, Cohort 51) of Ipilimumab and Nivolumab in Metastatic or Unresectable Angiosarcoma: A Substudy of Dual Anti-Ctla-4 and Anti-Pd-1 Blockade in Rare Tumors (Dart). *J Immunother Cancer* (2021) 9(8). doi: 10.1136/jitc-2021-002990.
15. Naqash AR, Coyne GHOS, Moore N, Sharon E, Takebe N, Fino KK, et al. Phase II Study of Atezolizumab in Advanced Alveolar Soft Part Sarcoma (Asps). *Journal of Clinical Oncology* (2021) 39(15_suppl):11519-. doi: 10.1200/JCO.2021.39.15_suppl.11519.
16. Smrke A OA, Napolitano A, Vergnano M, Asare B, Fotiadis N, et al., editor. 1526mo - Gemmk: A Phase I Study of Gemcitabine (Gem) and Pembrolizumab (Pem) in Patients (Pts) with Leiomyosarcoma (Lms) and Undifferentiated Pleomorphic Sarcoma Ups). *ESMO Congress 2021*; 2021: Annals of Oncology.
17. You Y, Guo X, Zhuang R, Zhang C, Wang Z, Shen F, et al. Activity of Pd-1 Inhibitor Combined with Anti-Angiogenic Therapy in Advanced Sarcoma: A Single-Center Retrospective Analysis. *Front Mol Biosci* (2021) 8:747650. Epub 20211116. doi: 10.3389/fmolb.2021.747650.
18. Li Y, Li HL, Hu HT, Shao SS, Chen CS, Guo CY, et al. Clinical Observation of Local Intervention Combined with Camrelizumab and Apatinib in the Treatment of Metastatic Soft-Tissue Sarcoma. *J Cancer Res Ther* (2021) 17(7):1718-24. doi: 10.4103/jcrt.jcrt_1310_21.
19. Wang J, Gao S, Yang Y, Liu X, Zhang P, Dong S, et al. Clinical Experience with Apatinib and Camrelizumab in Advance Clear Cell Sarcoma: A Retrospective Study. *Cancer Manag Res* (2021) 13:8999-9005. Epub 20211203. doi: 10.2147/CMAR.S337253.
20. Doshi A, Zhou M, Bui N, Wang DS, Ganjoo K, Hwang GL. Safety and Feasibility of Cryoablation During Immunotherapy in Patients with Metastatic Soft Tissue Sarcoma. *J Vasc Interv Radiol* (2021) 32(12):1688-94. Epub 20210831. doi: 10.1016/j.jvir.2021.08.017.
21. Scheinberg T, Lomax A, Tattersall M, Thomas D, McCowage G, Sullivan M, et al. Pd-1 Blockade Using Pembrolizumab in Adolescent and Young Adult Patients with Advanced Bone and Soft Tissue Sarcoma. *Cancer Rep (Hoboken)* (2021) 4(2):e1327. Epub 20201213. doi: 10.1002/cnr2.1327.
22. Starzer AM, Berghoff AS, Hamacher R, Tomasich E, Feldmann K, Hatziioannou T, et al. Tumor DNA Methylation Profiles Correlate with Response to Anti-Pd-1 Immune Checkpoint Inhibitor Monotherapy in Sarcoma Patients. *J Immunother Cancer* (2021) 9(3). doi: 10.1136/jitc-2020-001458.
23. Liu J, Fan Z, Bai C, Li S, Xue R, Gao T, et al. Real-World Experience with Pembrolizumab in Patients with Advanced Soft Tissue Sarcoma. *Ann Transl Med* (2021) 9(4):339. doi: 10.21037/atm-21-49.
24. Naing A, Thistlethwaite F, De Vries EGE, Eskens F, Uboha N, Ott PA, et al. Cx-072 (Pacmilimab), a Probody (®) Pd-L1 Inhibitor, in Advanced or Recurrent Solid Tumors (Proclaim-Cx-072): An Open-Label Dose-Finding and First-in-Human Study. *J Immunother Cancer* (2021) 9(7). doi: 10.1136/jitc-2021-002447.
25. Pollack SM, Redman MW, Baker KK, Wagner MJ, Schroeder BA, Loggers ET, et al. Assessment of Doxorubicin and Pembrolizumab in Patients with Advanced Anthracycline-Naive Sarcoma: A Phase 1/2 Nonrandomized Clinical Trial. *JAMA Oncol* (2020) 6(11):1778-82. doi: 10.1001/jamaoncol.2020.3689.
26. Martin-Broto J, Hindi N, Grignani G, Martinez-Trufero J, Redondo A, Valverde C, et al. Nivolumab and Sunitinib Combination in Advanced Soft Tissue Sarcomas: A Multicenter, Single-Arm, Phase Ib/II Trial. *J Immunother Cancer* (2020) 8(2). doi: 10.1136/jitc-2020-001561.

27. Roland CL, Keung EZ-Y, Lazar AJ, Torres KE, Wang W-L, Guadagnolo A, et al. Preliminary Results of a Phase II Study of Neoadjuvant Checkpoint Blockade for Surgically Resectable Undifferentiated Pleomorphic Sarcoma (Ups) and Dedifferentiated Liposarcoma (Ddlps). *Journal of Clinical Oncology* (2020) 38(15_suppl):11505-. doi: 10.1200/JCO.2020.38.15_suppl.11505.
28. Nathenson M, Choy E, Carr ND, Hibbard HD, Mazzola E, Catalano PJ, et al. Phase II Study of Eribulin and Pembrolizumab in Patients (Pts) with Metastatic Soft Tissue Sarcomas (Sts): Report of Lms Cohort. *Journal of Clinical Oncology* (2020) 38(15_suppl):11559-. doi: 10.1200/JCO.2020.38.15_suppl.11559.
29. Blay J CS, Penel N, Bertucci F, Bompas E, Saada-Bouzid E, et al. High Clinical Benefit Rates of Single Agent Pembrolizumab in Selected Rare Sarcoma Histotypes: First Results of the Acsé Pembrolizumab Study. *Annals of Oncology* (2020) 31, S972. doi: https://doi.org/10.1016/j.annonc.2020.08.1845.
30. Kawai A NT, Okamura N, Shibata T, Tamaura K, Ueda G. et al. Efficacy and Safety of Nivolumab Monotherapy in Patients with Unresectable Clear Cell Sarcoma and Alveolar Soft Part Sarcoma (Oscar Trial, Ncch1510): A Multicenter, Phase 2 Clinical Trial. *CTOS Virtual Annual Meeting* Vancouver, Canada(2020).
31. Naing A, Meric-Bernstam F, Stephen B, Karp DD, Hajjar J, Rodon Ahnert J, et al. Phase 2 Study of Pembrolizumab in Patients with Advanced Rare Cancers. *J Immunother Cancer* (2020) 8(1). doi: 10.1136/jitc-2019-000347.
32. Shi Y, Cai Q, Jiang Y, Huang G, Bi M, Wang B, et al. Activity and Safety of Geptanolimab (Gb226) for Patients with Unresectable, Recurrent, or Metastatic Alveolar Soft Part Sarcoma: A Phase II, Single-Arm Study. *Clin Cancer Res* (2020) 26(24):6445-52. Epub 20201012. doi: 10.1158/1078-0432.CCR-20-2819.
33. Naing A, Gainor JF, Gelderblom H, Forde PM, Butler MO, Lin CC, et al. A First-in-Human Phase 1 Dose Escalation Study of Spartalizumab (Pdr001), an Anti-Pd-1 Antibody, in Patients with Advanced Solid Tumors. *J Immunother Cancer* (2020) 8(1). doi: 10.1136/jitc-2020-000530.
34. Zhou M, Bui N, Bolleddu S, Lohman M, Becker HC, Ganjoo K. Nivolumab Plus Ipilimumab for Soft Tissue Sarcoma: A Single Institution Retrospective Review. *Immunotherapy* (2020) 12(18):1303-12. Epub 20200923. doi: 10.2217/imt-2020-0155.
35. Monga V, Skubitz KM, Maliske S, Mott SL, Dietz H, Hirbe AC, et al. A Retrospective Analysis of the Efficacy of Immunotherapy in Metastatic Soft-Tissue Sarcomas. *Cancers (Basel)* (2020) 12(7). Epub 20200711. doi: 10.3390/cancers12071873.
36. Ruohoniemi DM, Zhan C, Wei J, Kulkarni K, Aaltonen ET, Horn JC, et al. Safety and Effectiveness of Yttrium-90 Radioembolization around the Time of Immune Checkpoint Inhibitors for Unresectable Hepatic Metastases. *J Vasc Interv Radiol* (2020) 31(8):1233-41. doi: 10.1016/j.jvir.2020.04.029.
37. Yang J, Dong L, Yang S, Han X, Han Y, Jiang S, et al. Safety and Clinical Efficacy of Toripalimab, a Pd-1 Mab, in Patients with Advanced or Recurrent Malignancies in a Phase I Study. *Eur J Cancer* (2020) 130:182-92. Epub 20200327. doi: 10.1016/j.ejca.2020.01.028.
38. Quiroga D, Liebner DA, Philippon JS, Hoffman S, Tan Y, Chen JL, et al. Activity of Pd1 Inhibitor Therapy in Advanced Sarcoma: A Single-Center Retrospective Analysis. *BMC Cancer* (2020) 20(1):527. Epub 20200605. doi: 10.1186/s12885-020-07021-x.
39. MarjaNska A, Drogosiewicz M, Dembowska-BagiNska B, PawiNska WK, Balwierz W, Bobeff K, et al. Nivolumab for the Treatment of Advanced Pediatric Malignancies. *Anticancer Res* (2020) 40(12):7095-100. doi: 10.21873/anticanres.14738.

40. Le Cesne A, Marec-Berard P, Blay JY, Gaspar N, Bertucci F, Penel N, et al. Programmed Cell Death 1 (Pd-1) Targeting in Patients with Advanced Osteosarcomas: Results from the Pembrosarc Study. *Eur J Cancer* (2019) 119:151-7. Epub 20190821. doi: 10.1016/j.ejca.2019.07.018.
41. Wilky BA, Trucco MM, Subhawong TK, Florou V, Park W, Kwon D, et al. Axitinib Plus Pembrolizumab in Patients with Advanced Sarcomas Including Alveolar Soft-Part Sarcoma: A Single-Centre, Single-Arm, Phase 2 Trial. *Lancet Oncol* (2019) 20(6):837-48. Epub 20190508. doi: 10.1016/S1470-2045(19)30153-6.
42. Kelly CM, Chi P, Dickson MA, Gounder MM, Keohan ML, Qin L-X, et al. A Phase II Study of Epacadostat and Pembrolizumab in Patients with Advanced Sarcoma. *Journal of Clinical Oncology* (2019) 37(15_suppl):11049-. doi: 10.1200/JCO.2019.37.15_suppl.11049.
43. D'Angelo SP, Mahoney MR, Van Tine BA, Atkins J, Milhem MM, Jahagirdar BN, et al. Nivolumab with or without Ipilimumab Treatment for Metastatic Sarcoma (Alliance A091401): Two Open-Label, Non-Comparative, Randomised, Phase 2 Trials. *Lancet Oncol* (2018) 19(3):416-26. Epub 20180119. doi: 10.1016/s1470-2045(18)30006-8.
44. Gordon EM, Sankhala KK, Stumpf N, Ravicz J, Arasheben S, Leong B, et al. Cancer Immunotherapy Using Trabectedin and Nivolumab in Advanced Soft Tissue Sarcoma: A Retrospective Analysis. *Journal of Clinical Oncology* (2018) 36(5_suppl):40-. doi: 10.1200/JCO.2018.36.5_suppl.40.
45. Toulmonde M, Penel N, Adam J, Chevreau C, Blay JY, Le Cesne A, et al. Use of Pd-1 Targeting, Macrophage Infiltration, and Ido Pathway Activation in Sarcomas: A Phase 2 Clinical Trial. *JAMA Oncol* (2018) 4(1):93-7. doi: 10.1001/jamaoncol.2017.1617.
46. Weiss GJ, Waypa J, Blaydorn L, Coats J, McGahey K, Sangal A, et al. A Phase Ib Study of Pembrolizumab Plus Chemotherapy in Patients with Advanced Cancer (Pembroplus). *Br J Cancer* (2017) 117(1):33-40. Epub 20170606. doi: 10.1038/bjc.2017.145.
47. Ben-Ami E, Barysauskas CM, Solomon S, Tahlil K, Malley R, Hohos M, et al. Immunotherapy with Single Agent Nivolumab for Advanced Leiomyosarcoma of the Uterus: Results of a Phase 2 Study. *Cancer* (2017) 123(17):3285-90. Epub 20170425. doi: 10.1002/cncr.30738.
48. Tawbi HA, Burgess M, Bolejack V, Van Tine BA, Schuetze SM, Hu J, et al. Pembrolizumab in Advanced Soft-Tissue Sarcoma and Bone Sarcoma (Sarc028): A Multicentre, Two-Cohort, Single-Arm, Open-Label, Phase 2 Trial. *Lancet Oncol* (2017) 18(11):1493-501. Epub 20171004. doi: 10.1016/S1470-2045(17)30624-1.
49. D'Angelo SP, Shoushtari AN, Keohan ML, Dickson MA, Gounder MM, Chi P, et al. Combined Kit and Ctla-4 Blockade in Patients with Refractory Gist and Other Advanced Sarcomas: A Phase Ib Study of Dasatinib Plus Ipilimumab. *Clin Cancer Res* (2017) 23(12):2972-80. Epub 20161222. doi: 10.1158/1078-0432.CCR-16-2349.
50. Paoluzzi L, Cacavio A, Ghesani M, Karambelkar A, Rapkiewicz A, Weber J, et al. Response to Anti-Pd1 Therapy with Nivolumab in Metastatic Sarcomas. *Clin Sarcoma Res* (2016) 6:24. Epub 20161230. doi: 10.1186/s13569-016-0064-0.
51. Maki RG, Jungbluth AA, Gnjatic S, Schwartz GK, D'Adamo DR, Keohan ML, et al. A Pilot Study of Anti-Ctla4 Antibody Ipilimumab in Patients with Synovial Sarcoma. *Sarcoma* (2013) 2013:168145. Epub 20130227. doi: 10.1155/2013/168145.
52. D'Angelo SP, Druta M, Tine BAV, Liebner DA, Schuetze S, Nathenson M, et al. Primary Efficacy and Safety of Letetresgene Autoleucel (Lete-Cel; Gsk3377794) Pilot Study in Patients with Advanced and Metastatic Myxoid/Round Cell Liposarcoma (Mrcls). *Journal of Clinical Oncology* (2022) 40(16_suppl):11500-. doi: 10.1200/JCO.2022.40.16_suppl.11500.

53. Kohli K, Yao L, Nowicki TS, Zhang S, Black RG, Schroeder BA, et al. Il-15 Mediated Expansion of Rare Durable Memory T Cells Following Adoptive Cellular Therapy. *J Immunother Cancer* (2021) 9(5). doi: 10.1136/jitc-2020-002232.
54. Shi J, Li M, Yang R. Tumor-Infiltrating Lymphocytes as a Feasible Adjuvant Immunotherapy for Osteosarcoma with a Poor Response to Neoadjuvant Chemotherapy. *Immunotherapy* (2020) 12(9):641-52. Epub 20200603. doi: 10.2217/imt-2020-0107.
55. Zhou X, Wu J, Duan C, Liu Y. Retrospective Analysis of Adoptive Til Therapy Plus Anti-Pd1 Therapy in Patients with Chemotherapy-Resistant Metastatic Osteosarcoma. *J Immunol Res* (2020) 2020:7890985. Epub 20201001. doi: 10.1155/2020/7890985.
56. Wang C, Li M, Wei R, Wu J. Adoptive Transfer of Tils Plus Anti-Pd1 Therapy: An Alternative Combination Therapy for Treating Metastatic Osteosarcoma. *J Bone Oncol* (2020) 25:100332. Epub 20201016. doi: 10.1016/j.jbo.2020.100332.
57. Nowicki TS, Berent-Maoz B, Cheung-Lau G, Huang RR, Wang X, Tsoi J, et al. A Pilot Trial of the Combination of Transgenic Ny-Eso-1-Reactive Adoptive Cellular Therapy with Dendritic Cell Vaccination with or without Ipilimumab. *Clin Cancer Res* (2019) 25(7):2096-108. Epub 20181220. doi: 10.1158/1078-0432.Ccr-18-3496.
58. Ramachandran I, Lowther DE, Dryer-Minnerly R, Wang R, Fayngerts S, Nunez D, et al. Systemic and Local Immunity Following Adoptive Transfer of Ny-Eso-1 Spear T Cells in Synovial Sarcoma. *J Immunother Cancer* (2019) 7(1):276. Epub 20191024. doi: 10.1186/s40425-019-0762-2.
59. Van Tine BA, Butler MO, Araujo D, Johnson ML, Clarke J, Liebner D, et al., editors. 5471 - Adp-A2m4 (Mage-A4) in Patients with Synovial Sarcoma. *ESMO 2019 Congress* 2019: Annals of Oncology (2019).
60. Lu YC, Parker LL, Lu T, Zheng Z, Toomey MA, White DE, et al. Treatment of Patients with Metastatic Cancer Using a Major Histocompatibility Complex Class II-Restricted T-Cell Receptor Targeting the Cancer Germline Antigen Mage-A3. *J Clin Oncol* (2017) 35(29):3322-9. Epub 20170815. doi: 10.1200/jco.2017.74.5463.
61. Thiel U, Schober SJ, Einspieler I, Kirschner A, Thiede M, Schirmer D, et al. Ewing Sarcoma Partial Regression without Gvhd by Chondromodulin-I/Hla-a*02:01-Specific Allorestricted T Cell Receptor Transgenic T Cells. *Oncoimmunology* (2017) 6(5):e1312239. Epub 20170412. doi: 10.1080/2162402x.2017.1312239.
62. Ahmed N, Brawley VS, Hegde M, Robertson C, Ghazi A, Gerken C, et al. Human Epidermal Growth Factor Receptor 2 (Her2) -Specific Chimeric Antigen Receptor-Modified T Cells for the Immunotherapy of Her2-Positive Sarcoma. *J Clin Oncol* (2015) 33(15):1688-96. Epub 20150323. doi: 10.1200/JCO.2014.58.0225.
63. Robbins PF, Kassim SH, Tran TL, Crystal JS, Morgan RA, Feldman SA, et al. A Pilot Trial Using Lymphocytes Genetically Engineered with an Ny-Eso-1-Reactive T-Cell Receptor: Long-Term Follow-up and Correlates with Response. *Clin Cancer Res* (2015) 21(5):1019-27. Epub 20141223. doi: 10.1158/1078-0432.Ccr-14-2708.
64. Tonn T, Schwabe D, Klingemann HG, Becker S, Esser R, Koehl U, et al. Treatment of Patients with Advanced Cancer with the Natural Killer Cell Line Nk-92. *Cytotherapy* (2013) 15(12):1563-70. Epub 20131001. doi: 10.1016/j.jcyt.2013.06.017.
65. Montagna D, Turin I, Schiavo R, Montini E, Zaffaroni N, Villa R, et al. Feasibility and Safety of Adoptive Immunotherapy with Ex Vivo-Generated Autologous, Cytotoxic T Lymphocytes in Patients with Solid Tumor. *Cytotherapy* (2012) 14(1):80-90. Epub 20110923. doi: 10.3109/14653249.2011.610303.

66. Dillman RO, Soori G, DePriest C, Nayak SK, Beutel LD, Schiltz PM, et al. Treatment of Human Solid Malignancies with Autologous Activated Lymphocytes and Cimetidine: A Phase II Trial of the Cancer Biotherapy Research Group. *Cancer Biother Radiopharm* (2003) 18(5):727-33. doi: 10.1089/108497803770418274.
67. Mazumder A, Eberlein TJ, Grimm EA, Wilson DJ, Keenan AM, Aamodt R, et al. Phase I Study of the Adoptive Immunotherapy of Human Cancer with Lectin Activated Autologous Mononuclear Cells. *Cancer* (1984) 53(4):896-905. doi: 10.1002/1097-0142(19840215)53:4<896::aid-cncr2820530414>3.0.co;2-e.
68. DeMaria PJ, Bilusic M, Park DM, Heery CR, Donahue RN, Madan RA, et al. Randomized, Double-Blind, Placebo-Controlled Phase II Study of Yeast-Brachyury Vaccine (Gi-6301) in Combination with Standard-of-Care Radiotherapy in Locally Advanced, Unresectable Chordoma. *Oncologist* (2021) 26(5):e847-e58. Epub 20210309. doi: 10.1002/onco.13720.
69. Dhir A, Koru-Sengul T, Grosso J, D'Amato GZ, Trucco MM, Rosenberg A, et al. Phase 1 Trial of Autologous Dendritic Cell Vaccination with Imiquimod Immunomodulation in Children and Adults with Refractory Sarcoma. *Journal of Clinical Oncology* (2021) 39(15_suppl):11542-. doi: 10.1200/JCO.2021.39.15_suppl.11542.
70. Somaiah N, Chawla SP, Block MS, Morris JC, Do K, Kim JW, et al. A Phase 1b Study Evaluating the Safety, Tolerability, and Immunogenicity of Cmb305, a Lentiviral-Based Prime-Boost Vaccine Regimen, in Patients with Locally Advanced, Relapsed, or Metastatic Cancer Expressing Ny-Eso-1. *Oncoimmunology* (2020) 9(1):1847846. Epub 20201119. doi: 10.1080/2162402X.2020.1847846.
71. Kelly CM, Antonescu CR, Bowler T, Munhoz R, Chi P, Dickson MA, et al. Objective Response Rate among Patients with Locally Advanced or Metastatic Sarcoma Treated with Talimogene Laherparepvec in Combination with Pembrolizumab: A Phase 2 Clinical Trial. *JAMA Oncol* (2020) 6(3):402-8. doi: 10.1001/jamaoncol.2019.6152.
72. Fedorova L, Mudry P, Pilatova K, Selingerova I, Merhautova J, Rehak Z, et al. Assessment of Immune Response Following Dendritic Cell-Based Immunotherapy in Pediatric Patients with Relapsing Sarcoma. *Front Oncol* (2019) 9:1169. Epub 20191114. doi: 10.3389/fonc.2019.01169.
73. Miwa S, Nishida H, Tanzawa Y, Takeuchi A, Hayashi K, Yamamoto N, et al. Phase 1/2 Study of Immunotherapy with Dendritic Cells Pulsed with Autologous Tumor Lysate in Patients with Refractory Bone and Soft Tissue Sarcoma. *Cancer* (2017) 123(9):1576-84. Epub 20170227. doi: 10.1002/cncr.30606.
74. Merchant MS, Bernstein D, Amoako M, Baird K, Fleisher TA, Morre M, et al. Adjuvant Immunotherapy to Improve Outcome in High-Risk Pediatric Sarcomas. *Clin Cancer Res* (2016) 22(13):3182-91. Epub 20160128. doi: 10.1158/1078-0432.CCR-15-2550.
75. Ghisoli M, Barve M, Mennel R, Lenarsky C, Horvath S, Wallraven G, et al. Three-Year Follow up of Gmcsf/Bi-Shrna(Furin) DNA-Transfected Autologous Tumor Immunotherapy (Vigil) in Metastatic Advanced Ewing's Sarcoma. *Mol Ther* (2016) 24(8):1478-83. Epub 20160425. doi: 10.1038/mt.2016.86.
76. Krishnadas DK, Shusterman S, Bai F, Diller L, Sullivan JE, Cheerva AC, et al. A Phase I Trial Combining Decitabine/Dendritic Cell Vaccine Targeting Mage-A1, Mage-A3 and Ny-Eso-1 for Children with Relapsed or Therapy-Refractory Neuroblastoma and Sarcoma. *Cancer Immunol Immunother* (2015) 64(10):1251-60. Epub 20150624. doi: 10.1007/s00262-015-1731-3.
77. Cripe TP, Ngo MC, Geller JI, Louis CU, Currier MA, Racadio JM, et al. Phase 1 Study of Intratumoral Pexa-Vec (Jx-594), an Oncolytic and Immunotherapeutic Vaccinia Virus, in Pediatric Cancer Patients. *Mol Ther* (2015) 23(3):602-8. Epub 20141222. doi: 10.1038/mt.2014.243.

78. Takahashi R, Ishibashi Y, Hiraoka K, Matsueda S, Kawano K, Kawahara A, et al. Phase II Study of Personalized Peptide Vaccination for Refractory Bone and Soft Tissue Sarcoma Patients. *Cancer Sci* (2013) 104(10):1285-94. Epub 20130806. doi: 10.1111/cas.12226.
79. Coosemans A, Vanderstraeten A, Tuyaerts S, Verschuere T, Moerman P, Berneman ZN, et al. Wilms' Tumor Gene 1 (Wt1)--Loaded Dendritic Cell Immunotherapy in Patients with Uterine Tumors: A Phase I/II Clinical Trial. *Anticancer Res* (2013) 33(12):5495-500.
80. Himoudi N, Wallace R, Parsley KL, Gilmour K, Barrie AU, Howe K, et al. Lack of T-Cell Responses Following Autologous Tumour Lysate Pulsed Dendritic Cell Vaccination, in Patients with Relapsed Osteosarcoma. *Clin Transl Oncol* (2012) 14(4):271-9. doi: 10.1007/s12094-012-0795-1.
81. Karbach J, Gnjatic S, Bender A, Neumann A, Weidmann E, Yuan J, et al. Tumor-Reactive Cd8+ T-Cell Responses after Vaccination with Ny-Eso-1 Peptide, Cpg 7909 and Montanide Isa-51: Association with Survival. *Int J Cancer* (2010) 126(4):909-18. doi: 10.1002/ijc.24850.
82. Suminoe A, Matsuzaki A, Hattori H, Koga Y, Hara T. Immunotherapy with Autologous Dendritic Cells and Tumor Antigens for Children with Refractory Malignant Solid Tumors. *Pediatr Transplant* (2009) 13(6):746-53. Epub 20081101. doi: 10.1111/j.1399-3046.2008.01066.x.
83. Dillman R, Barth N, Selvan S, Beutel L, de Leon C, DePriest C, et al. Phase I/II Trial of Autologous Tumor Cell Line-Derived Vaccines for Recurrent or Metastatic Sarcomas. *Cancer Biother Radiopharm* (2004) 19(5):581-8. doi: 10.1089/cbr.2004.19.581.
84. Hernando JJ, Park TW, Kubler K, Offergeld R, Schlebusch H, Bauknecht T. Vaccination with Autologous Tumour Antigen-Pulsed Dendritic Cells in Advanced Gynaecological Malignancies: Clinical and Immunological Evaluation of a Phase I Trial. *Cancer Immunol Immunother* (2002) 51(1):45-52. Epub 20020110. doi: 10.1007/s00262-001-0255-1.
85. Geiger JD, Hutchinson RJ, Hohenkirk LF, McKenna EA, Yanik GA, Levine JE, et al. Vaccination of Pediatric Solid Tumor Patients with Tumor Lysate-Pulsed Dendritic Cells Can Expand Specific T Cells and Mediate Tumor Regression. *Cancer Res* (2001) 61(23):8513-9.
86. Campanacci M, Bacci G, Bertoni F, Picci P, Minutillo A, Franceschi C. The Treatment of Osteosarcoma of the Extremities: Twenty Year's Experience at the Istituto Ortopedico Rizzoli. *Cancer* (1981) 48(7):1569-81. doi: 10.1002/1097-0142(19811001)48:7<1569::aid-cncr2820480717>3.0.co;2-x.
87. Zhang S, Kohli K, Black RG, Yao L, Spadinger SM, He Q, et al. Systemic Interferon-Gamma Increases Mhc Class I Expression and T-Cell Infiltration in Cold Tumors: Results of a Phase 0 Clinical Trial. *Cancer Immunol Res* (2019) 7(8):1237-43. Epub 20190606. doi: 10.1158/2326-6066.CIR-18-0940.
88. Meazza C, Cefalo G, Massimino M, Daolio P, Pastorino U, Scanagatta P, et al. Primary Metastatic Osteosarcoma: Results of a Prospective Study in Children Given Chemotherapy and Interleukin-2. *Med Oncol* (2017) 34(12):191. Epub 20171101. doi: 10.1007/s12032-017-1052-9.
89. Miki Y, Tada T, Kamo R, Hosono MN, Tamiya H, Shimatani Y, et al. Single Institutional Experience of the Treatment of Angiosarcoma of the Face and Scalp. *Br J Radiol* (2013) 86(1030):20130439. doi: 10.1259/bjr.20130439.
90. Ogawa K, Takahashi K, Asato Y, Yamamoto Y, Taira K, Matori S, et al. Treatment and Prognosis of Angiosarcoma of the Scalp and Face: A Retrospective Analysis of 48 Patients. *Br J Radiol* (2012) 85(1019):e1127-33. Epub 20120717. doi: 10.1259/bjr/31655219.
91. Ohguri T, Imada H, Nomoto S, Yahara K, Hisaoka M, Hashimoto H, et al. Angiosarcoma of the Scalp Treated with Curative Radiotherapy Plus Recombinant Interleukin-2 Immunotherapy. *Int J Radiat Oncol Biol Phys* (2005) 61(5):1446-53. doi: 10.1016/j.ijrobp.2004.08.008.

92. Sasaki R, Soejima T, Kishi K, Imajo Y, Hirota S, Kamikonya N, et al. Angiosarcoma Treated with Radiotherapy: Impact of Tumor Type and Size on Outcome. *Int J Radiat Oncol Biol Phys* (2002) 52(4):1032-40. doi: 10.1016/s0360-3016(01)02753-5.
93. Le Cesne A, Vassal G, Farace F, Spielmann M, Le Chevalier T, Angevin E, et al. Combination Interleukin-2 and Doxorubicin in Advanced Adult Solid Tumors: Circumvention of Doxorubicin Resistance in Soft-Tissue Sarcoma? *J Immunother* (1999) 22(3):268-77. doi: 10.1097/00002371-199905000-00010.
